# m^6^A methyltransferase METTL3 programs CD4^+^ T-cell activation and effector T-cell differentiation in systemic lupus erythematosus

**DOI:** 10.1186/s10020-023-00643-4

**Published:** 2023-04-03

**Authors:** Shuang Lu, Xingyu Wei, Huan Zhu, Zhi Hu, Meiling Zheng, Jiali Wu, Cheng Zhao, Shuang Yang, Delong Feng, Sujie Jia, Hongjun Zhao, Ming Zhao

**Affiliations:** 1 Department of Dermatology, Hunan Key Laboratory of Medical Epigenomics, The Second Xiangya Hospital, Central South University, Changsha, 410011 China; 2 Department of Pharmacy, The Third Xiangya Hospital, Central South University, Changsha, 410013 China; 3 Department of Rheumatology, Xiangya Hospital, Central South University, Changsha, 410008 China; 4 Institute of Dermatology, Chinese Academy of Medical Sciences and Peking Union Medical College, Nanjing, 210042 China

**Keywords:** *N*6-Methyladenosine, METTL3, SLE, Autoimmune disorders, mRNA methylation

## Abstract

**Background:**

Systemic lupus erythematosus (SLE) is an autoimmune disorder in which excessive CD4^+^ T-cell activation and imbalanced effector T-cell differentiation play critical roles. Recent studies have implied a potential association between posttranscriptional *N6*-methyladenosine (m^6^A) modification and CD4^+^ T-cell-mediated humoral immunity. However, how this biological process contributes to lupus is not well understood. In this work, we investigated the role of the m^6^A methyltransferase like 3 (METTL3) in CD4^+^ T-cell activation, differentiation, and SLE pathogenesis both in vitro and in vivo.

**Methods:**

The expression of METTL3 was knocked down and METTL3 enzyme activity was inhibited using siRNA and catalytic inhibitor, respectively. In vivo evaluation of METTL3 inhibition on CD4^+^ T-cell activation, effector T-cell differentiation, and SLE pathogenesis was achieved using a sheep red blood cell (SRBC)-immunized mouse model and a chronic graft versus host disease (cGVHD) mouse model. RNA-seq was performed to identify pathways and gene signatures targeted by METTL3. m^6^A RNA-immunoprecipitation qPCR was applied to confirm the m^6^A modification of METTL3 targets.

**Results:**

METTL3 was defective in the CD4^+^ T cells of SLE patients. METTL3 expression varied following CD4^+^ T-cell activation and effector T-cell differentiation in vitro. Pharmacological inhibition of METTL3 promoted the activation of CD4^+^ T cells and influenced the differentiation of effector T cells, predominantly Treg cells, in vivo. Moreover, METTL3 inhibition increased antibody production and aggravated the lupus-like phenotype in cGVHD mice. Further investigation revealed that catalytic inhibition of METTL3 reduced Foxp3 expression by enhancing Foxp3 mRNA decay in a m^6^A-dependent manner, hence suppressing Treg cell differentiation.

**Conclusion:**

In summary, our findings demonstrated that METTL3 was required for stabilizing Foxp3 mRNA via m^6^A modification to maintain the Treg differentiation program. METTL3 inhibition contributed to the pathogenesis of SLE by participating in the activation of CD4^+^ T cells and imbalance of effector T-cell differentiation, which could serve as a potential target for therapeutic intervention in SLE.

**Supplementary Information:**

The online version contains supplementary material available at 10.1186/s10020-023-00643-4.

## Introduction

Systemic lupus erythematosus (SLE) is an autoimmune disease characterized by an overactivated immune system, sustained autoantibody production, and accumulation of immune complexes that eventually causes dysfunction of multiple organs and systems (Carter et al. 2016). Excessive activation of CD4^+^ T lymphocytes and effector T-cell differentiation abnormalities, which contributed to disease pathophysiology, have been described in SLE patients (Li et al. 2022). Inflammatory cytokines secreted by effector CD4^+^ T lymphocytes, including IL-4, IL-17, and IFN-γ, also play an essential role in the inflammatory response of SLE (Moulton and Tsokos 2015).

RNA m^6^A modification has been reported to regulate effector T-cell differentiation and function (Tong et al. 2018; Zhou et al. 2021). m^6^A is involved in RNA splicing, elongation, decay, and translation. m^6^A decoration is dynamically regulated by methyltransferases (writers), demethylases (erasers), and m^6^A binding proteins (readers). METTL3 is the predominant catalytic subunit of the methyltransferase complex, with methyltransferase like protein 14 (METTL14) assisting with substrate recognition (Wang et al. 2016; Liu et al. 2014). In addition, Wilms tumor 1-associating protein (WTAP) guides the nuclear localization of the METTL3-METTL14 complex (Ping et al. 2014). So far, the only two erasers identified include ALKB homolog 5 (ALKBH5) and fat mass and obesity-associated protein (FTO). ALKBH5-mediated m^6^A demethylation regulates mRNA stability and export to the cytoplasm (Zheng et al. 2013; Li et al. 2019). The demethylation activity of FTO on m^6^A is more pronounced in the nucleus (Wei et al. 2018), and the removal of the m^6^A group by FTO modulates RNA alternative splicing by preventing the binding of splicing factor 2 to splice sites (Zhao et al. 2014). Recent studies have identified METTL14 and ALKBH5 as differentially expressed genes in SLE peripheral blood mononuclear cells (PBMCs) compared with those in healthy controls (HCs) (Luo et al. 2020). Another study also reported a downregulated expression of METTL3, WTAP, and FTO in the peripheral blood of SLE patients, suggesting a potential link between m^6^A modification and SLE pathogenesis (Luo et al. 2020). However, how m^6^A enzymes dynamically affect CD4^+^ T-cell homeostasis and function in SLE has not been well explored.

In this work, we found that METTL3 expression was significantly downregulated in the peripheral CD4^+^ T cells of SLE patients, and was negatively correlated with SLE disease activity. METTL3 expression was markedly altered in in vitro-induced activated and differentiated CD4^+^ T cells. METTL3 inhibition by STM2457 (Yankova et al. 2021), a highly potent and selective catalytic inhibitor of METTL3, significantly promoted CD4^+^ T-cell activation and reprogrammed effector T-cell differentiation in mice. Additionally, pharmacological inhibition of METTL3 enhanced autoantibody production and exacerbated the lupus-like phenotype in cGVHD mice. Further investigation revealed that METTL3 promoted Treg differentiation by regulating Foxp3 expression in a m^6^A-dependent manner, demonstrating a potential role of METTL3 in the pathogenesis of SLE.

## Methods

### Patients and healthy subjects

Patients who met the 2012 Systemic Lupus Collaborating Clinics for SLE, the 2002 American-European Consensus Group for primary Sjogren's syndrome (pSS), the 2010 ACR/EULAR for rheumatoid arthritis (RA), and criteria based on pathologic examination for psoriasis (PS) were recruited from the Second Xiangya Hospital. Age and gender-matched healthy donors from medical staff were enrolled for comparison. Information on HCs and patients with an autoimmune disorder is listed in Additional file 1.

### In vitro human CD4^+^ T-cell isolation and culture

Total CD4^+^ T cells were isolated from PBMCs using human CD4 microbeads (Miltenyi Biotec, Germany), and naïve CD4^+^ T cells were selected by human Naïve CD4^+^ T Cell Isolation Kit (Miltenyi Biotec). Purified cells were then cultured in RPMI 1640 medium (GIBCO, USA) supplemented with 10% fetal bovine serum (GIBCO), and 1% penicillin/streptomycin (Beyotime, China) at 37 °C with 5% CO_2_.

### In vitro human naïve CD4^+^ T-cell activation and differentiation

Human naïve CD4^+^ T cells were activated and differentiated under certain conditions. Briefly, cells were cultured under the stimulation of precoated anti-CD3 antibody (2 μg/ml, Sigma-Aldrich, USA) and anti-CD28 antibody (1 μg/ml, Sigma-Aldrich) for the desired time with the intention of activation. In addition to anti-CD3 and anti-CD28 antibodies, human naïve CD4^+^ T cells were cultured under the following polarization conditions for cell differentiation: anti-IL-4 antibody (10 μg/ml, Peprotech, USA), IL-12 (10 ng/ml, Peprotech), IL-2 (5 ng/ml, Peprotech) for Th1 polarization; anti-IFN-γ antibody (10 μg/ml, Peprotech), IL-2 (5 ng/ml), IL-4 (25 ng/ml, Peprotech) for Th2 polarization; anti-IFN-γ antibody (10 μg/ml), anti-IL-4 antibody (10 μg/ml), IL-6 (25 ng/ml, Peprotech), TGF-β (5 ng/ml, R&D System, USA), IL-1β (12.5 ng/ml, Peprotech), IL-21 (20 ng/ml, R&D System), IL-23 (25 ng/ml, Peprotech) for Th17 polarization; IL-6 (20 ng/ml), IL-21 (20 ng/ml), IL-12 (10 ng/ml), TGF-β (5 ng/ml) for Tfh polarization, IL-2 (10 ng/ml), TGF-β (5 ng/ml) for Treg polarization.

### Flow cytometry

Single-cell suspensions were incubated with fluorescently labeled antibodies according to the manufacturer’s protocols, and then analyzed (FACS Canto II, BD Biosciences, Canada) or sorted (FACS Arial II, BD Biosciences) by flow cytometry. Data were collected and analyzed by FlowJo-V10 software. All antibodies used are listed in Additional file 2.

### Quantitative real-time PCR

Total RNAs were obtained using the TRIzol reagent (MRC, USA), reverse transcribed using the PrimeScript RT Kit (Takara, Japan), and quantified by quantitative real-time PCR using SYBR Green reagents (Bio-Rad, USA) according to the manufacturer’s instructions. The primer sequences are provided in Additional file 3.

### siRNA transfection

METTL3 siRNA or scrambled siRNA was transfected into naïve CD4^+^ T cells using the Human T Cell Nucleofector Kit and Amaxa Nucleofector system (Lonza, USA). siRNA sequences can be found in Additional file 3.

### RNA-sequencing

Differentiated Treg cells were treated with either STM2457 (Selleck, USA, 5 μM) or DMSO for 5 days. Total RNA was extracted and submitted to Novo-gene for RNA sequencing. Sequencing data and experimental protocols were submitted to the National Center for Biotechnology Information (NCBI) Gene Expression Omnibus (GEO) (GSE213483). Differentially expressed genes between the two groups were screened and filtered by a *p*-value of less than 0.05. log2FoldChange values from RNA-seq analysis were plotted for the two groups for Treg-related gene sets to create a heatmap.

### meRIP (m^6^A immunoprecipitation)-qPCR

meRIP-qPCR was performed using the Methylated RNA Immunoprecipitation Kit (Sigma-Aldrich) according to the manufacturer’s protocols. In brief, RNA was extracted from CD4^+^ T-cell samples using TRIzol reagent and fragmented using fragmentation buffer. The fragmented RNA samples were then incubated with anti-m^6^A antibody or rabbit IgG in the immunoprecipitating buffer. m^6^A-RNA complexes were enriched using protein A/G beads and purified by wash buffer, followed by RNA elution using elution buffer. RNA was subjected to reverse transcription and qPCR using the PrimeScript RT Kit (Takara) and SYBR Green reagents (Bio-rad), respectively, according to the manufacturer’s instructions. The enrichment of the PCR product in the target RNA fragment was compared to the % of RNA quantity in the input sample. Primer sequences for meRIP-qPCR are supplied in Additional file 3.

### Western blot

For the preparation of cellular lysates, cells were harvested with immunoprecipitation assay buffer (Beyotime) supplemented with a proteinase inhibitor cocktail (Roche, USA) and phenylmethanesulfonyl fluoride (Sigma-Aldrich). Protein concentration was determined using the Pierce BCA Protein Assay Kit (Thermo Fisher Scientific, USA). Protein samples were heat denatured and separated by sodium dodecyl sulfate (SDS)-PAGE gel-electrophoresis and transferred to PVDF membranes (Millipore, USA). After blocking in phosphate-buffered saline (PBS)/Tween-20 containing 5% nonfat milk, the membranes were incubated with primary antibodies overnight at 4 °C, followed by incubation with HRP-conjugated anti-rabbit/mouse secondary IgG antibodies (CST, USA) for an hour. Then the protein bands were visualized using SuperSignal West Pico PLUS (Thermo Fisher Scientific) and analyzed by ImageQuant LAS 4000 mini (GE-Healthcare, USA). The information for primary antibodies is presented in Additional file 4. Original blots can be found in Additional file 5.

### RNA m^6^A quantification

Total RNA was isolated using TRIzol reagent according to the manufacturer’s instructions. Global m^6^A levels in total RNA were evaluated by the EpiQuick™ m6A RNA Methylation Quantification Kit (Colorimetric) (EPIGENTEK, USA). In brief, 200 ng of total RNA per reaction was optimally prepared and bound to each well. The capture antibody was added after several washes, and then the detection antibody, enhancer solution, and color-developing solution were added successively for color development. The m^6^A levels were quantified by measuring the absorbance of each well at a wavelength of 450 nm based on the standard curve.

### RNA degradation assay

CD4^+^ T cells from HCs were isolated and induced Treg polarization in vitro after STM2457 or DMSO treatment. Actinomycin D (Sigma-Aldrich) was added to each well at a final concentration of 10 μM. Cells were then harvested at 0, 2, and 6 h after adding actinomycin D and processed with qRT-PCR to determine the relative residual mRNA level. Data were normalized to the t = 0 time point.

### SRBC immunization and STM2457 administration

Six-week-old C57/BL6J female mice were purchased from Slack Company (Shanghai, China) and immunized intraperitoneally with 500 μl of 5% SRBCs twice at an interval of 1 week. After the 2nd SRBC injection, STM2457, or DMSO control was intraperitoneally administered at 30 mg/kg/day for 1 week until the mice were harvested. After the mice were sacrificed by anesthesia, splenic leukocytes were isolated and prepared into a single-cell suspension by erythrocyte lysis with red cell lysis buffer (Thermo Fisher Scientific).

### cGVHD lupus mouse model

DBA2 mice and B2D6F1 mice were purchased from SPF company (Beijing, China). A total of 5 × 10^7^ lymphocytes (spleen: thymus: lymph nodes = 3:2:1) from DBA2 mice were injected into the tail vein of B2D6F1 mice four times to create a cGVHD lupus mouse model. STM2457 (30 mg/kg) or DMSO control was intraperitoneally administered once every 3 days 1 week after the last injection of lymphocytes. Mice were sacrificed at 10 weeks. Kidney tissues were collected, fixed, and embedded in paraffin. H&E staining was performed on kidney sections using a standard protocol. Deposition of IgG and C3 in the kidneys was visualized by IHC staining.

### ELISA

Mouse anti-SRBC antibody, total IgG, dsDNA, ANA (CUSABIO, USA), IFN-γ, and IL-17A (Thermo Fisher Scientific) in the serum were measured by ELISA according to the manufacturer’s instructions. Briefly, samples or standards were added to the precoated well and incubated for 2 h at 37 °C. Subsequently, biotin-antibody was added to each well and incubated for 1 h at 37 °C, followed by incubation with HRP-avidin for another hour. TMB substrate was added and incubated for 15 min at 37 °C for signal development, and then the plate was read at 450 nm immediately after the addition of Stop Solution. Information on secondary antibodies is provided in Additional file 4.

### Statistics

ANOVA models and unpaired Student’s *t* test were used to compare the group differences, and Pearson’s correlation was applied for the correlation analysis. All data are presented as mean ± SD unless otherwise noted. Statistical significance was calculated as described in each figure.

## Results

### METTL3 is downregulated in peripheral CD4^+^ T cells of SLE patients

**Fig. 1:**
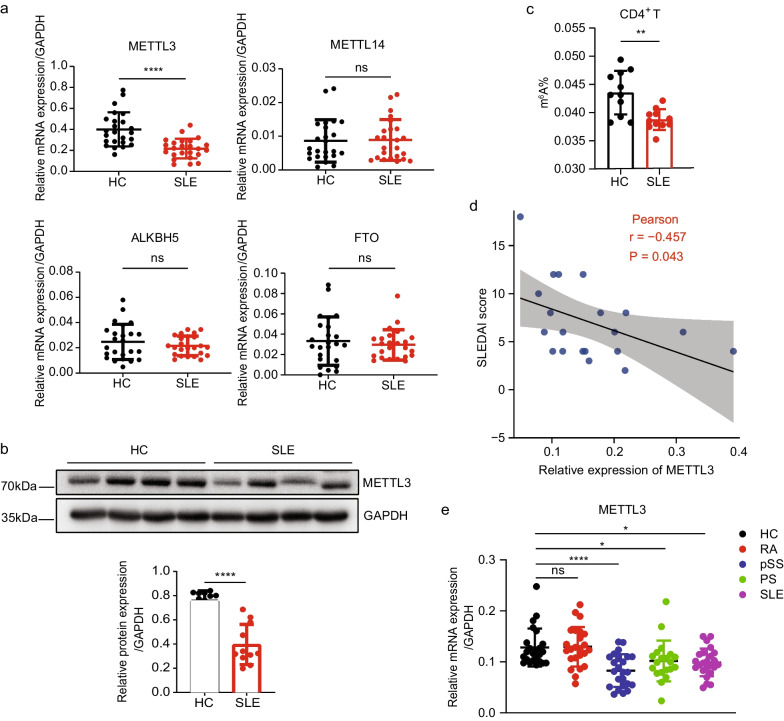
METTL3 expression is significantly reduced in SLE CD4^+^ T cells compared with healthy controls (HCs). **a** Quantitative reverse transcription polymerase chain reaction (RT-qPCR) of the mRNA expression of METTL3, METTL14, ALKBH5, and FTO in SLE CD4^+^ T cells relative to HCs, n = 24. **b** Top: METTL3 protein levels in CD4^+^ T cells of SLE patients and HCs were detected by Western blot analysis, and GAPDH was used as a loading control; bottom: quantification of METTL3 protein levels, n = 11. **c** Global m^6^A modification level in CD4^+^ T cells was determined by m^6^A colorimetric quantification in SLE patients compared with HCs, n = 11. **d** Correlation between the SLEDAI score and mRNA expression of METTL3 in SLE CD4^+^ T cells, n = 20. **e** RT-qPCR of METTL3 expression in rheumatoid arthritis (RA), primary Sjogren’s Syndrome (pSS), psoriasis (PS), and SLE compared with HCs, n = 24. (*p < 0.05, **p < 0.01, ****p < 0.0001, ns, no significance, unpaired two-tailed Student’s *t* test for **a**–**c**, Pearson’s correlation analysis for **d**, one-way ANOVA with Dunnett’s multiple comparisons test for **e**)

We first investigated the expression of m^6^A modification enzymes in peripheral CD4^+^ T cells of SLE patients. qRT-PCR analysis showed that the mRNA expression of METTL3 in SLE CD4^+^ T cells was significantly decreased compared with that in HCs, but no significant difference in the expression of METTL14, ALKBH5, and FTO was observed (Fig. 1a). Western blot confirmed the reduced protein level of METTL3 in SLE CD4^+^ T cells (Fig. 1b). In line with the downregulated expression of the m^6^A writer, the global m^6^A level was also reduced in SLE CD4^+^ T cells (Fig. 1c). Furthermore, correlation analysis indicated that METTL3 mRNA expression in CD4^+^ T cells was negatively correlated with SLE disease activity index (SLEDAI) scores, suggesting a potential role of METTL3 in the pathogenesis of SLE (Fig. 1d). Comparison of METTL3 mRNA expression in CD4^+^ T cells among various autoimmune disorders relative to HCs demonstrated that, except for RA, METTL3 expression was generally decreased in SLE, pSS, and PS, further prompting the essentiality of METTL3 in CD4^+^ T-cell-mediated autoimmune and inflammatory responses (Fig. 1e).

### The expression of METTL3 is altered during CD4^+^ T-cell activation and effector T-cell differentiation

**Fig. 2:**
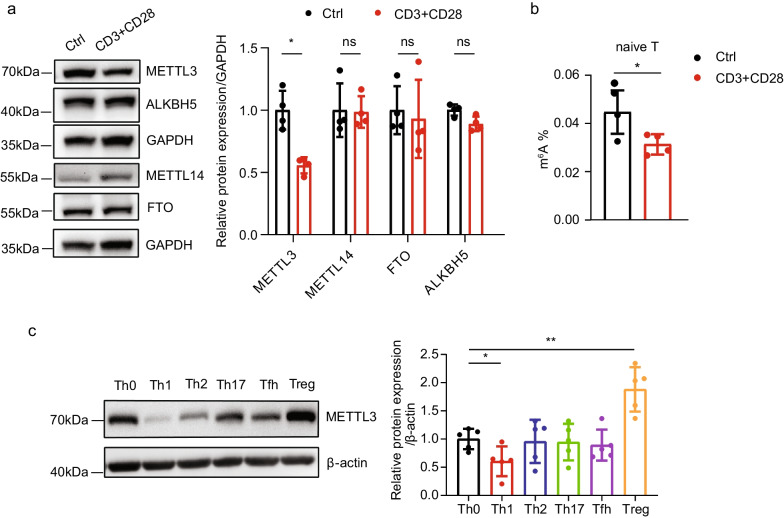
METTL3 expression varies after inducing T-cell activation and differentiation in vitro. **a** Left: the protein levels of the m^6^A-modifying enzymes METTL3, METTL14, ALKBH5, and FTO in naïve CD4^+^ T cells activated by anti-CD3 and anti-CD28 antibodies relative to control cells were determined by Western blot analysis. GAPDH was used as a loading control; right: quantification of protein levels, n = 4. **b** Total m^6^A modification level in naïve CD4^+^ T cells before and after activation was examined by m^6^A colorimetric quantification, n = 4. **c** Left: Western blot analysis of METTL3 protein expression among polarized Th1, Th2, Th17, Tfh, and Treg cell subsets relative to Th0, and β-actin was used as a loading control; right: quantification of METTL3 protein expression among different T-cell subsets, n = 5. (*p < 0.05, **p < 0.01, ns, no significance, two-way ANOVA with Sidak’s multiple comparisons test for **a**, unpaired two-tailed Student’s *t* test for **b**, one-way ANOVA with Dunnett’s multiple comparisons test for **c**)

Given that the excessive activation of CD4^+^ T cells is an essential mechanism during SLE pathogenesis (Moulton and Tsokos 2015; Mak and Kow 2014), naïve CD4^+^ T cells were isolated from HCs and were induced activation in vitro to evaluate the possible involvement of m^6^A in CD4^+^ T-cell behavior. Activated CD4^+^ T cells displayed significantly reduced expression of METTL3 at both the mRNA and protein levels, whereas no significant change was noted in the expression of other writers and erasers (Fig. 2a, Additional file 6: Fig. S1a, b). In line with the downregulated expression of METTL3, the global m^6^A level in CD4^+^ T cells was also reduced after cell activation (Fig. 2b). We next induced distinct effector T-cell differentiation in vitro as previously indicated (Wu et al. 2018). METTL3 expression was significantly decreased in the Th1 subset and elevated in the Treg subset, indicating that METTL3 might play context-dependent roles during effector T-cell differentiation (Fig. 2c, Additional file 6: Fig. S1c).

### Pharmacological inhibition of METTL3 regulates T-cell activation and differentiation during the humoral immune response in vivo

**Fig. 3:**
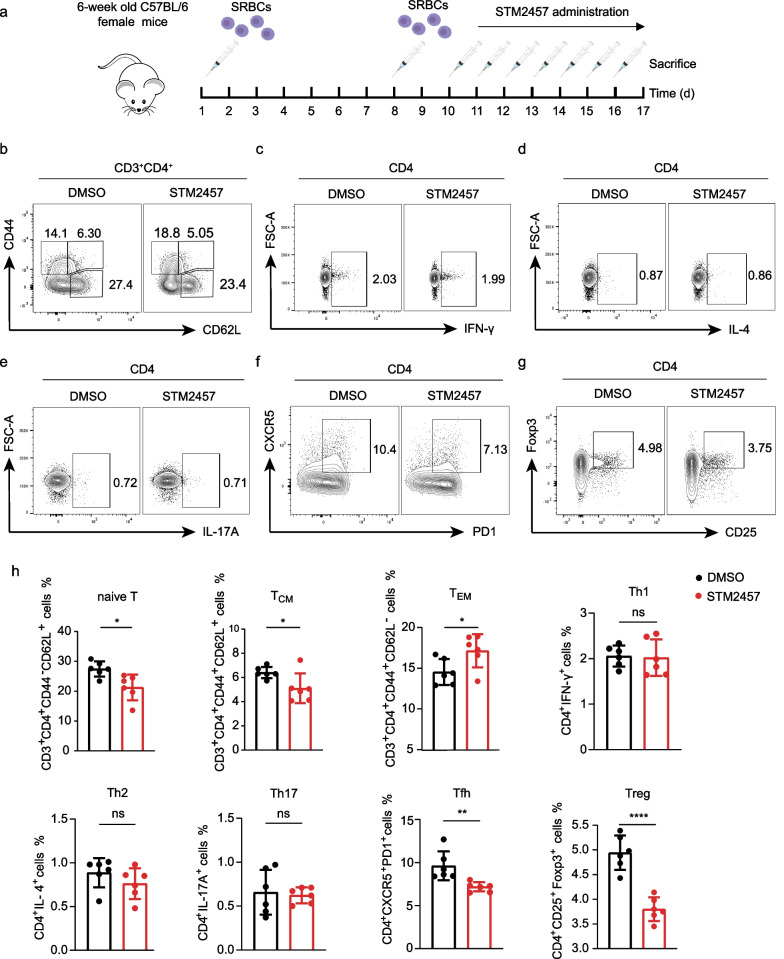
Inhibition of METTL3 activity promotes CD4^+^ T-cell activation and suppresses Tfh and Treg cell differentiation in vivo. **a** Schematic diagram of SRBC immunization and STM2457 administration. STM2457 is a highly potent and selective catalytic inhibitor of MELLT3. **b**–**g** Representative dot plots showing the proportions of CD3^+^CD4^+^CD44^−^CD62L^+^ naïve T cells, CD3^+^CD4^+^CD44^+^CD62L^+^ T_CM_ cells, CD3^+^CD4^+^CD44^+^CD62L^−^ T_EM_ cells (**b**), CD4^+^IFN-γ^+^ Th1 cells (**c**), CD4^+^IL-4^+ ^Th2 cells (**d**), CD4^+^IL-17A^+ ^Th17 cells (**e**), CD4^+^PD1^+^CXCR5^+^ Tfh cells (**f**), and CD4^+^CD25^+^Foxp3^+^ Treg cells (**g**) in the spleen of STM2457-treated and DMSO control mice after SRBC challenge; T_CM_, central memory T cells; T_EM_, effective memory T cells. **h** Quantification plots of splenic T cells, n = 6. Data are representative of 2 independent experiments. (*p < 0.05, **p < 0.01, ****p < 0.0001, ns, no significance, unpaired two-tailed Student’s *t* test)

To evaluate the effects of METTL3 on CD4^+^ T-cell activation and differentiation in vivo, we intraperitoneally immunized C57BL/6J mice with SRBCs that could independently stimulate T-cell-dependent humoral immunity (Fig. 3a). The immunized mice were then administered with STM2457, a selective catalytic inhibitor of MELLT3, or DMSO control. As expected, the serum levels of anti-SRBC IgM, IgG1, IgG2a, IgG2b, IgG (H+L), and IgG3 were markedly increased after SRBC challenge (Additional file 6: Fig. S2a). SRBC-immunized mice also had significantly elevated splenic Tfh recruitment (Additional file 6: Fig. S2b), indicating satisfactory SRBC modeling. We investigated whether the dysfunction of METTL3 enzyme activity affected CD4^+^ T-cell activation in vivo by assessing the frequencies of naïve T cells, central memory T cells (T_CM_), and effective memory T cells (T_EM_). METTL3 catalytic inhibition significantly reduced proportions of naïve T and T_CM_ cells, and elevated T_EM_ cell frequency (Fig. 3b, h), representing enhanced splenic CD4^+^ T-cell activation. Mice subjected to STM2457 displayed comparable proportions of IFN-γ^+^ (Fig. 3c, h), IL-4^+^ (Fig. 3d, h), and IL-17A^+^ (Fig. 3e, h) CD4^+^ T cells relative to the DMSO control group. However, significantly reduced frequencies of splenic CD4^+^PD1^+^CXCR5^+^ Tfh cells (Fig. 3f, h) and CD4^+^CD25^+^Foxp3^+^ Treg cells (Fig. 3g, h) were observed in mice administered with STM2457, indicating suppressed differentiation of Tfh and Treg cells by METTL3 catalytic inhibition. Of note, the suppressed proportion of Tfh cells agrees with a previous study, where conditional knockout of METTL3 in CD4^+^ T cells impaired Tfh cell differentiation of mice (Yao et al. 2021). Taken together, these data suggest that the pharmacological inhibition of METTL3 in SRBC-immunized mice significantly promotes T-cell activation and attenuates Tfh and Treg cell differentiation.

### Pharmacological inhibition of METTL3 aggravates the lupus-like phenotype in a cGVHD mouse model

**Fig. 4:**
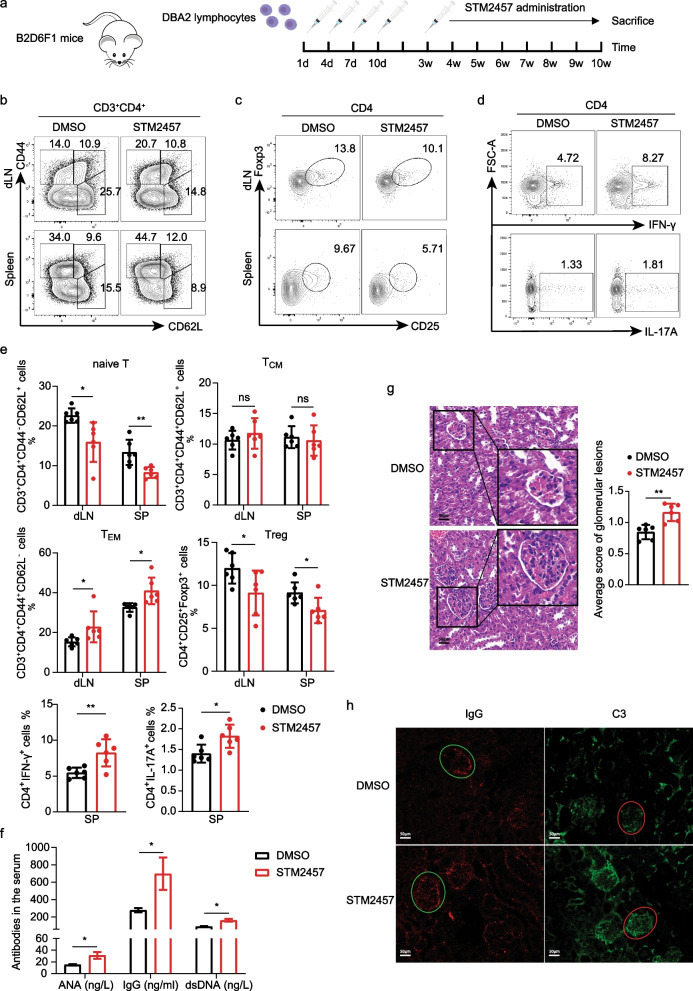
Pharmacological inhibition of METTL3 aggravates the lupus-like phenotype in a cGVHD mouse model. **a** Schematic diagram of the cGVHD model and STM2457 administration. **b**, **c** Representative dot plots showing the proportions of naïve T cells, T_CM_ cells, T_EM_ cells (**b**), and Treg cells (**c**) in the spleen and dLNs of cGVHD mice. **d** Representative dot plots showing the proportions of splenic Th1 and Th17 cells in cGVHD mice. **e** Quantification plots of cells from **b** to **d**, n = 6. Data are representative of 2 independent experiments. **f** ELISA comparing the ANA, IgG, and dsDNA antibodies in the serum of cGVHD mice treated with either DMSO or STM2457 (mean ± SEM). **g** Representative H&E staining images of kidney sections and the grades of glomerular lesions of cGVHD mice treated with either DMSO or STM2457. Scale bar: 25 μm. **h** Representative histological images showing C3 and IgG deposition in the kidneys of cGVHD mice treated with either DMSO or STM2457. Scale bar: 50 μm. (*p < 0.05, **p < 0.01, ns, no significance, unpaired two-tailed Student’s *t* test)

cGVHD is characterized by systemic autoimmunity similar to human SLE (Gleichmann et al. 1982). To assess the role of METTL3 in the pathogenesis of lupus, we generated a cGVHD mouse model by tail vein injection of lymphocytes from DBA2 mice into B6D2F1 mice, followed by intraperitoneal administration of STM2457 or DMSO control (Fig. 4a). STM2457 administration did not cause a noticeable change in mouse body weight (Additional file 6: Fig. S3a) or the size of the spleen and draining lymph nodes (dLNs) (Additional file 6: Fig. S3b). In parallel with the observations in the SRBC model, STM2457 administration in the cGVHD model also led to a significantly reduced proportion of naïve T cells and an elevated frequency of T_EM_ cells in both the spleen and dLNs (Fig. 4b, e), indicative of promoted T-cell activation. Compared with control mice, mice that received STM2457 treatment had similar proportions of Tfh cells in both the spleen and dLNs (Additional file 6: Fig. S3c), and the splenic Th2 subtype (Additional file 6: Fig. S3d), but showed marked suppression of the frequency of Treg cells (Fig. 4c, e) and elevation of splenic Th1 and Th17 proportions (Fig. 4d, e), collectively suggesting a potential proinflammatory effect of METTL3 inhibition on cGVHD mice. Although the Th1 and Th17 frequencies were promoted, the IFN-γ and IL-17A cytokines in the serum remained comparable between the two groups (Additional file 6: Fig. S3e), which may be attributed to the relatively low percentages of Th1 and Th17 subtypes.

We next sought to determine the ANA, IgG, and dsDNA autoantibodies in the serum by ELISA. Mice subjected to STM2457 treatment exhibited significantly elevated production of all three antibodies at week 10 (Fig. 4f). In addition, STM2457-treated mice developed more severe renal damage than control mice, as shown by enhanced lymphocyte infiltration and higher renal scores based on H&E staining (Fig. 4g). In line with this observation, histological analysis showed elevated deposition of renal C3 and IgG immune complexes in STM2457-treated mice (Fig. 4h). In summary, these data support that catalytic inhibition of METTL3 contributes to autoantibody production and the lupus-like phenotype in cGVHD mice, potentially by promoting CD4^+^ T cell activation and affecting the imbalanced differentiation of effector T cells.

### Pharmacological inhibition of METTL3 restrains the Treg gene signature

To confirm our findings that METTL3 inhibition promotes CD4^+^ T-cell activation in mice, human naïve CD4^+^ T cells were treated with either STM2457 or DMSO before inducing cell activation. As expected, STM2457 treatment significantly reduced the global m^6^A level (Additional file 6: Fig. S4a). The proportion of CD4^+^CD25^−^CD69^−^ cells, which represent unactivated cells, was also decreased after METTL3 inhibition (Additional file 6: Fig. S4b). We then knocked down METTL3 expression by siRNA (Additional file 6: Fig. S4c) and found that, in parallel with STM2457 treatment, METTL3 gene depletion also accelerated CD4^+^ T-cell activation (Additional file 6: Fig. S4d), further confirming the essentiality of METTL3 in suppressing CD4^+^ T-cell activation.

**Fig. 5:**
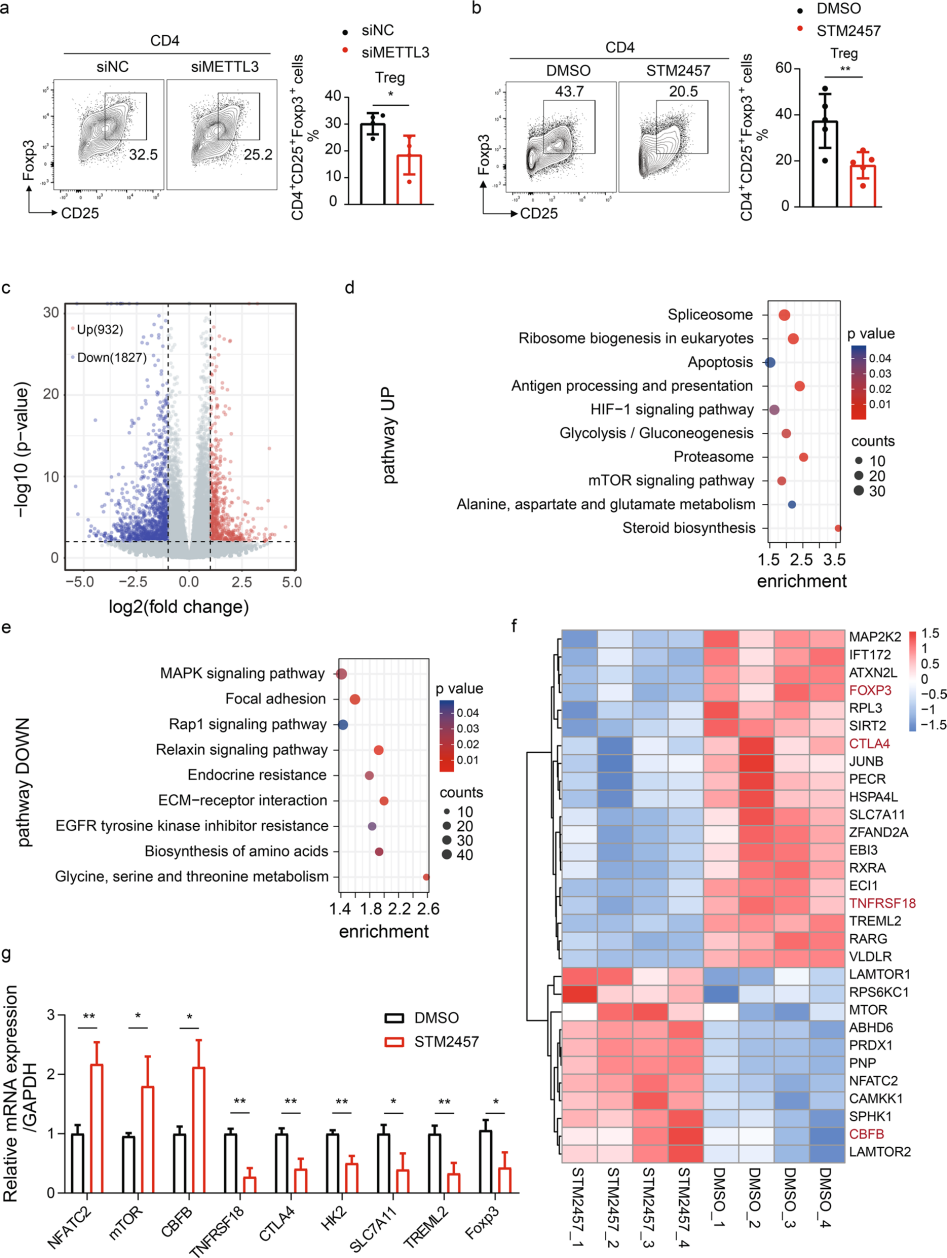
Inhibition of METTL3 activity induces epigenetic reprogramming of differentiated Treg cells. **a** Left: Representative dot plots showing the proportion of CD4^+^ T cells treated with either scramble siRNA or METTL3 siRNA while cultured under Treg differentiation conditions for 5 days; right: quantification of CD4^+^CD25^+^Foxp3^+^ Treg cells, n = 4. **b** Left: representative dot plots showing the composition of CD4^+^ T cells in the STM2457-treated and DMSO control groups under Treg differentiation conditions for 5 days; right: quantification of Treg cells, n = 5. **c** Volcano plot of differentially expressed genes in the STM2457-and DMSO-treated CD4^+^ T cells isolated from human peripheral blood and cultured under Treg differentiation conditions for 5 days. |log_2_foldchange| > 1, *p* < 0.05; red, upregulated genes; blue, downregulated genes; four biological replicates for each group were used for analysis. **d**, **e** KEGG pathway enrichment analysis of upregulated (**d**) and downregulated (**e**) genes in STM2457-treated CD4^+^ T cells compared with control cells. **f** Clustered heatmap of 30 Treg signature genes altered after the inhibition of METTL3. **g** RT-qPCR analysis validating the differentially expressed Treg signature gene transcripts identified by RNA-seq, n = 3. (*p < 0.05, **p < 0.01, unpaired two-tailed Student’s *t* test for **a** and **b**, one-way ANOVA with Dunnett’s multiple comparisons test for **g**)

Following our observations that in vitro differentiated Treg cells expressed the highest level of METTL3 compared with other Th subsets (Fig. 2c), and that the inhibition of METTL3 significantly attenuated the Treg cell proportion in mice (Figs. 3 and 4), we hypothesized that METTL3 plays an indispensable role in Treg cell differentiation. Indeed, METTL3 gene knockdown by siRNA or catalytic inhibition by STM2457 consistently suppressed in vitro Treg cell differentiation (Fig. 5a, b), suggesting a m^6^A-dependent pro-differentiative effect of METTL3 on Treg cells.

To investigate the mechanism by which METTL3 regulates Treg cell differentiation, we performed RNA-seq analysis of induced Treg cells treated with either DMSO or STM2457. A total of 2759 differentially expressed genes were identified (fold change > 2 or < 0.5, *p* < 0.05), among which, 932 genes were upregulated, whereas 1827 genes were downregulated after METTL3 inhibition (Fig. 5c). Kyoto Encyclopedia of Genes and Genomes (KEGG) showed that the top upregulated pathways included some of the critical pathways that were linked to the inhibition of Treg cell differentiation, including the mTOR signaling pathway (Delgoffe et al. 2009; Essig et al. 2017) and HIF-1 signaling pathway (Dang et al. 2011; Shi et al. 2011) (Fig. 5d). The top downregulated pathways included the MAPK and Rap1 pathways, which are associated with the promotion of Treg differentiation (Huang et al. 2018; Ishihara et al. 2022) (Fig. 5e). Taken together, these profoundly altered pathways induced by catalytic inhibition of METTL3 might cooperatively contribute to the restriction of Treg cell differentiation.

To gain insight into specific genes targeted by METTL3, we queried the Gene Set Enrichment Analysis (GSEA) database for specific Treg gene signatures and compared their expression between Treg cells exposed to DMSO or STM2457. Genes positively correlated with Treg cell differentiation, like CTLA4 (Sakaguchi et al. 2020), TNFRSF18 (Mijnheer et al. 2021), and FOXP3, were significantly downregulated. In contrast, MTOR (Huang et al. 2020) and CBFB (Kitoh et al. 2009), which were negatively associated with Treg cell differentiation, were upregulated (Fig. 5f). RT-qPCR further confirmed the altered expression of Treg signature genes that were potential m^6^A targets regulated by METTL3 (Fig. 5g).

### Catalytic inhibition of METTL3 reduces Foxp3 expression by decreasing its m^6^A modification and mRNA stability

**Fig. 6:**
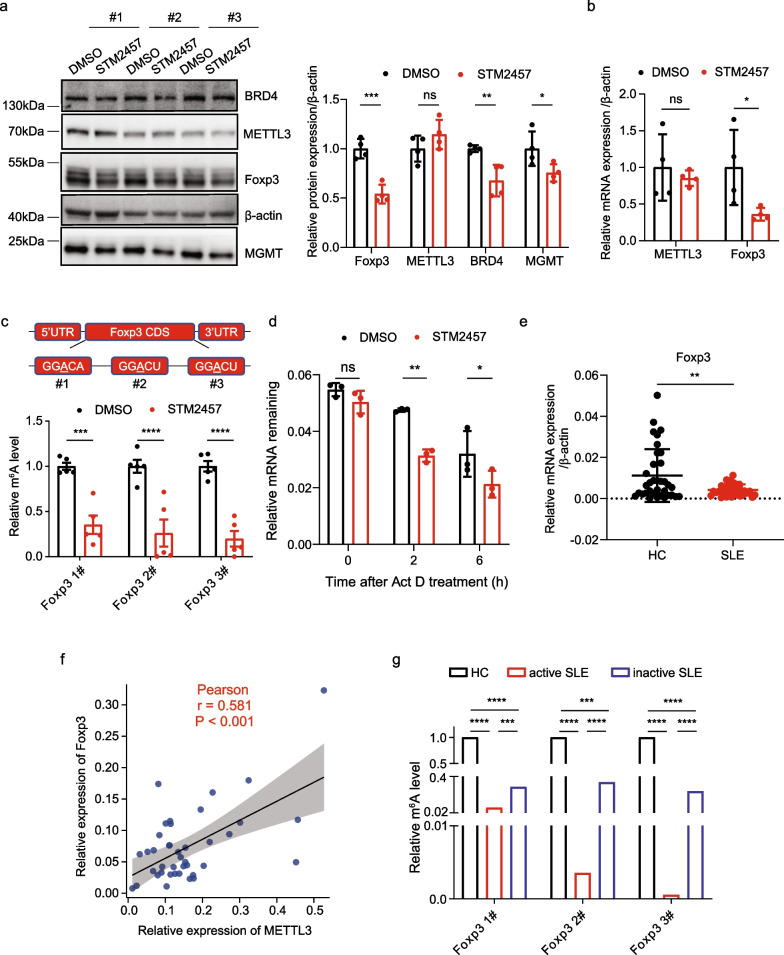
Inhibition of METTL3 activity reduces Foxp3 mRNA stability and expression by decreasing m^6^A modification. **a** Left: Foxp3 protein expression was determined by Western blot in CD4^+^ T cells treated with either DMSO or STM2457 when cultured under Treg differentiation conditions. BRD4 and MGMT were used as positive controls, and β-actin was used as a loading reference; right: quantification of protein bands, n = 4. **b** RT-qPCR of METTL3 and Foxp3 mRNA in flow-sorted CD4^+^CD25^+^CD127^−^ cells after treatment with either DMSO or STM2457 when cultured under Treg differentiation conditions. **c** Top: predicted m^6^A modification sites in the CDS of Foxp3; bottom: meRIP-qPCR (m^6^A immunoprecipitation-qPCR) comparing the m^6^A enrichment on three predicted m^6^A sites of Foxp3 mRNA in DMSO- or STM2457-treated CD4^+^ T cells. The results are presented as a relative percentage normalized to inputs, n = 5. **d** Degradation of Foxp3 mRNA was measured in differentiated Treg cells treated with either DMSO or STM2457 after the addition of actinomycin D for 0, 2, and 6 h. The results were normalized to 0 h, n = 4. **e** RT-qPCR of Foxp3 mRNA in CD4^+^ T cells of SLE patients and HCs, n = 24. **f** Correlation analysis of the mRNA expression of METTL3 and Foxp3 in SLE CD4^+^ T cells, n = 40. **g** meRIP-qPCR of m^6^A enrichment at three predicted m^6^A sites in Foxp3 mRNA in CD4^+^ T cells isolated from HC, patients with active SLE, and patients with inactive SLE. Inactive SLE: SLEDAI ≤ 4, active SLE: SLEDAI > 4. The results are presented as relative percentages normalized to inputs. (*p < 0.05, **p < 0.01, ***p < 0.001, ****p < 0.0001, ns, no significance, two-way ANOVA with Sidak’s multiple comparisons test for **a**, **c** and **d**, unpaired two-tailed Student’s *t* test for **b** and **e**, Pearson’s correlation analysis for **f**, and Chi-square test for **g**)

Given the importance of Foxp3 in Treg cell differentiation, we sought to determine whether Foxp3 is a m^6^A target gene directly regulated by METTL3. STM2457 treatment did not cause a visible change in METTL3 expression, but significantly suppressed Foxp3 expression (Fig. 6a). Flow-sorted Tregs gated on CD4^+^CD25^+^CD127^−^ cells also showed similar results for METTL3 and Foxp3 expression (Fig. 6b; Additional file 6: Fig. S4e). As positive controls, BRD4 (Choe et al. 2018) and MGMT (Shi et al. 2021), two previously reported METTL3 targets, indeed decreased after STM2457 treatment (Fig. 6a). Moreover, METTL3 expression in SLE CD4^+^ T cells was indistinguishable between the DMSO and STM2457-treated groups (Additional file 6: Fig. S4f), indicating that STM2457 particularly inhibits the activity of METTL3 without apparently affecting its expression during Treg cell differentiation. To confirm whether METTL3 inhibition reduces Foxp3 expression by affecting its m^6^A deposition, meRIP-qPCR was performed and revealed that the m^6^A abundance of all the three predicted m^6^A-tagged sites (Zhou et al. 2016) on Foxp3 mRNA was significantly decreased after METTL3 inhibition. This implies that Foxp3 is a potential m^6^A target directly regulated by METTL3 (Fig. 6c). Given that m^6^A modification of mRNA primarily affects mRNA stability (Zaccara et al. 2019), we performed an RNA decay assay via actinomycin D (ActD) treatment to evaluate the stability of Foxp3 mRNA after METTL3 inhibition. Foxp3 mRNA in cells subjected to STM2457 exhibited a substantially accelerated decrease compared to the control group at different time points after ActD treatment (Fig. 6d). These observations collectively suggest that METTL3 promotes Treg cell differentiation predominantly by enhancing Foxp3 mRNA stability.

### m^6^A level of Foxp3 mRNA was reduced in CD4^+^ T cells of SLE

We next detected Foxp3 expression in CD4^+^ T cells from both SLE patients and HCs. The mRNA level of Foxp3 was significantly reduced in SLE patients compared to HCs (Fig. 6e), which was positively correlated with METTL3 expression, further supporting the tight regulation of Foxp3 by METTL3 (Fig. 6f). Thereafter, the m^6^A level of Foxp3 in CD4^+^ T cells of patients with active SLE or inactive SLE was quantified using meRIP-qPCR. Due to the requirement of a large RNA amount for IP reaction and limited access to SLE blood samples, a pooled sample for each group was prepared for IP reaction, and a Chi-square test was applied for statistical analysis. Foxp3 mRNA in SLE patients exhibited uniformly reduced m^6^A deposition at all three indicated loci, which was consistent with the observation that METTL3 positively regulated the expression of Foxp3 in a m^6^A-dependent manner (Fig. 6g). Interestingly, Foxp3 mRNA from patients with active SLE possessed even less m^6^A than that from patients with inactive SLE, indicating a potential association between Foxp3 m^6^A modification and SLE activity.

## Discussion

Although emerging evidence supports the indispensability of epigenetic alterations in SLE pathogenesis, our interpretation of how RNA m^6^A modification affects SLE pathogenesis is still elementary. Recent studies have indicated that multiple m^6^A enzymes are differentially expressed in the peripheral blood of SLE patients (Luo et al. 2020; Luo et al. 2020). Whether and to what extent dysregulation of m^6^A modification is involved in SLE remains to be determined. Here, we found that SLE CD4^+^ T cells had significantly lower METTL3 abundance and subsequently that its expression was altered upon T-cell activation and distinct effector T-cell differentiation. Catalytic inhibition of METTL3 led to promoted activation of CD4^+^ T cells, elevated frequencies of Th1 and Th17 subsets, and impaired differentiation of Treg cells in mice. These observations suggested a preference and asymmetry effect of METTL3 in T-cell activation and differentiation.

One recent study demonstrated that METTL3 depletion in mouse T cells disrupted T-cell homeostasis and differentiation by enhancing the mRNA stability of inhibitory SOCS family genes via m^6^A (Li et al. 2017). We observed that pharmacological inhibition of METTL3 by STM2457 promoted CD4^+^ T-cell activation and reduced the population of Treg cells in a humoral SRBC model and lupus-like cGVHD model. siRNA-mediated gene knockdown and catalytic inhibition of METTL3 yielded similar effects, strongly confirming an m^6^A-dependent mechanism of METTL3 on CD4^+^ T activation and differentiation. Despite being unaffected in SRBC-immunized mice, the populations of Th1 and Th17 cells in cGVHD mice significantly increased by STM2457 treatment, indicating the critical roles of METTL3 in anti-inflammatory responses and maintenance of effector T-cell balance.

Treg cells, as regulators of peripheral immunological tolerance, are essential for preventing the development of autoimmune diseases (Dominguez-Villar and Hafler 2018). Another group noted that METTL3 is required for the suppressive function of Treg cells (Tong et al. 2018). This research, along with our findings, collectively revealed the irreplaceability of METTL3 in Treg cell differentiation and function. However, they did not observe a significant alteration in the Treg population after conditional knockout of METTL3 in naïve T cells (Li et al. 2017). The variation we observed in Treg expansion could be attributed to the different cell species and distinct experimental approaches adopted. siRNA-mediated gene knockdown or catalytic inhibition of enzyme activity represents a short-term impact of METTL3 loss on cell behavior. Furthermore, pharmacological inhibition of METTL3 by STM2457 preserves the integrity of the METTL3-METTL14 methyltransferase complex, hence avoiding potential unexpected distractions resulting from enzyme structure disruption.

The functional outcomes of m^6^A modification in transcripts highly depend on specific m^6^A regions modulated by different enzymes and recognized by different readers (Shi et al. 2019). A recent study reported that m^6^A modification in the 3′UTR of TCF7 enhanced its mRNA stability (Yao et al. 2021). Our finding that METTL3 inhibition promotes Foxp3 mRNA degradation by reducing mRNA m^6^A deposition further supports an mRNA-stabilizing role of m^6^A modification mediated by METTL3.

Recent studies have shown the implications of METTL3 in immune responses and inflammation regulation (Li et al. 2021; Wang et al. 2019; Winkler et al. 2019). Decreased m^6^A levels in SARS-CoV-2 and host genes by METTL3 depletion enhance the innate immune signaling pathway and inflammatory gene expression (Li et al. 2021). Specific METTL3 deletion in dendritic cells impairs the phenotypic and functional maturation of DCs in a m^6^A-dependent manner (Wang et al. 2019). METTL3-mediated m^6^A modification controls the innate immune response by targeting type I interferons (Winkler et al. 2019). Nevertheless, the mechanisms by which METTL3 dysregulation participates in the initiation and progression of SLE remains unclear. Pharmacological inhibition of METTL3 in our cGVHD model significantly aggravated the lupus-like phenotype, as indicated by higher levels of autoantibodies in the serum, more severe kidney damage, and more accumulation of immune complexes in the glomerulus. The comparison of METTL3 mRNA levels in peripheral CD4^+^ T cells revealed universally reduced expression of METTL3 in multiple autoimmune disorders, indicating the potentially crucial role of METTL3 in CD4^+^ T-cell function during autoimmune responses. However, whether our findings can be generalized to other autoimmune diseases warrant further investigation.

In summary, we uncovered an essential role of METTL3 in regulating T-cell activation, effector T-cell differentiation, and the lupus phenotype in SLE. METTL3-mediated m^6^A modification of Foxp3 mRNA stabilizes the transcript and maintains Foxp3 protein expression to facilitate Treg cell differentiation.

## Conclusion

In conclusion, our study highlights the significance of the m^6^A-directed posttranscriptional mechanism of METTL3 in CD4^+^ T-cell activation and differentiation during systemic autoimmune responses, revealing METTL3 as a potential novel target in SLE for therapeutic purposes.

## Supplementary Information

**Additional file 1: Table S1.** Information on HCs and patients.

**Additional file 2: Table S2.** Information on materials and reagents for Flow analysis.

**Additional file 3: Table S3.** Sequence information on siRNA and primers

**Additional file 4: Table S4.** Antibody information for Western blot and ELISA

**Additional file 5.** Original blots of Western blot analysis.

**Additional file 6: Figure S1.** METTL3 expression varies after T-cell activation and Treg-cell differentiation in vitro. **a** Representative dot plots showing the proportions of CD4^+^ T cells before and after activation by anti-CD3 and anti-CD28 antibodies for 3 days. **b** The mRNA expression of METTL3 in CD4^+^ T cells activated by anti-CD3 and anti-CD28 antibodies at 0, 12, 48, and 72 h was detected by RT-qPCR, n = 3. **c** Left: Western blot of METTL3 expression in Th0 and differentiated Tregs at 3 and 5 days, and β-actin was used as a loading control; right: quantification of METTL3 protein expression in Th0 and differentiated Tregs, n = 3. (***p < 0.001, ns, no significance, one-way ANOVA with Dunnett’s multiple comparisons test for **b**, two-way ANOVA with Sidak’s multiple comparisons test for **c**). **Figure S2.** Anti-SRBC antibodies and splenic Tfh cells are significantly elevated in SRBC-immunized mice. **a** ELISA of anti-SRBC specific antibodies IgM, IgG1, IgG2a, IgG2b, IgG (H+L), and IgG3 in the blood serum of control mice and SRBC-immunized mice. Serum dilution: IgM: 1:200; IgG1, IgG2a, and IgG (H+L): 1:400; IgG2b: 1:800; IgG3: 1:40; n = 6. **b** Left: representative dot plots showing the proportion of splenic CD4^+^ T cells in mice after SRBC challenge; right: quantification of splenic CD4^+^PD1^+^CXCR5^+^ Tfh cells, n = 6. (*p < 0.05, **p < 0.01, ***p < 0.001 ****p < 0.0001, unpaired two-tailed Student’s *t* test). **Figure S3.** Proportions of Tfh and Th2 cells remain comparable between control and STM2457-administered cGVHD mice. **a** Comparison of the body weights of cGVHD mice body treated with DMSO or STM2457. **b** Left: spleen and dLN images of cGVHD mice; right: quantification of spleen weight. **c** Top: representative dot plots showing the proportion of CD4^+^ T cells in the spleen and dLNs of cGVHD mice treated with DMSO or STM2457; bottom: quantification of CD4^+^PD1^+^CXCR5^+^ Tfh cells. **d** Left: representative dot plots showing the proportion of splenic CD4^+^ T cells in the spleens of cGVHD mice treated with DMSO or STM2457; right: quantification of CD4^+^IL-4^+^ Th2 cells. **e** ELISA of IFN-γ and IL-17A levels in the blood serum of cGVHD mice treated with DMSO or STM2457. (ns, no significance, unpaired two-tailed Student’s *t* test). **Figure S4.** METTL3 catalytic inhibition or gene knockdown suppresses CD4^+^ T-cell activation in vitro. **a** Comparison of total m^6^A modification between STM2457- and DMSO-treated CD4^+^ T cells by colorimetric quantification, n = 5. **b** Left: representative dot plots showing the composition of CD4^+^ T cells in STM2457 (5 μM)-treated and DMSO control cells after activation by anti-CD3 and anti-CD28 antibodies for 3 days; right: quantification of nonactivated CD4^+^CD25^−^CD69^−^ cells, n = 5. **c** Immunoblot of METTL3 in CD4^+^ T cells treated with either METTL3 siRNA or scramble siRNA, and β-actin was used as a loading control.** d** Left: representative dot plots showing the proportion of CD4^+^ T cells treated with either scramble siRNA or METTL3 siRNA for 3 days after being activated by anti-CD3 and anti-CD28 antibodies; right: quantification of unactivated CD4^+^CD25^−^CD69^−^ cells, n = 3. **e** Left: representative dot plots showing the proportion of CD4^+^ T cells treated with either DMSO or STM2457 for 5 days when cultured under Treg differentiation conditions; right: quantification of CD4^+^CD25^+^CD127^−^ cells, n = 4. **f** The mRNA expression of METTL3 in SLE CD4^+^ T cells treated with either DMSO or STM2457 when cultured under Treg differentiation conditions was detected by RT-qPCR, n = 4. (*p < 0.05, **p < 0.01, ***p < 0.001, ns, no significance, unpaired two-tailed Student’s *t* test).

## Data Availability

The dataset supporting the conclusions of this article is available in the NCBI GEO (GSE213483, https://www.ncbi.nlm.nih.gov/geo/).

## Abbreviations

SLE: Systemic lupus erythematosus
m^6^A: *N* (6)-Methyladenosine
METTL3: methyltransferase like protein 3
SRBC: Sheep red blood cell
cGVHD: Chronic graft versus host disease
ALKBH5: ALKB homolog 5
METTL14: methyltransferase like protein 14
WTAP: Wilms tomor 1-associated protein
ALKBH5: ALKB homolog 5
FTO: Fat mass and obesity-associated protein
PBMC: Peripheral blood mononuclear cell
HC: Healthy control
NCBI: National Center for Biotechnology Information
GEO: Gene Expression Omnibus
meRIP: M^6^A immunoprecipitation
RA: Rheumatoid arthritis
pSS: Primary Sjogren’s syndrome
PS: Psoriasis
SLEDAI: SLE disease activity index
KEGG: Kyoto Encyclopedia of Genes and Genomes
GSEA: Gene Set Enrichment Analysis
ActD: Actinomycin D
T_CM_: Central memory T cell
T_EM_: Effective memory T cell
dLNs: Draining lymph nodes
