# Supplementary material for: m6A methyltransferase METTL3 programs CD4+ T-cell activation and effector T-cell differentiation in systemic lupus erythematosus
Source: Mol Med. 2023 Apr 3;29:46. doi: 10.1186/s10020-023-00643-4 (PMC10068720; doi:10.1186/s10020-023-00643-4)
Supplement: Supplementary file 1 — Additional file 1: Table S1. Information on HCs and patients. [file 10020_2023_643_MOESM1_ESM.docx]

**Table S1. Information on HCs and patients**

| Identifier | Gender | Age | SLEDAI score |
| --- | --- | --- | --- |
| SLE1 | Male | 33 | 12 |
| SLE2 | Female | 21 | 8 |
| SLE3 | Female | 55 | 2 |
| SLE4 | Female | 37 | 12 |
| SLE5 | Female | 48 | 10 |
| SLE6 | Female | 59 | 18 |
| SLE7 | Female | 51 | 4 |
| SLE8 | Female | 33 | 4 |
| SLE9 | Male | 35 | 8 |
| SLE10 | Female | 54 | 6 |
| SLE11 | Female | 39 | 6 |
| SLE12 | Female | 37 | 4 |
| SLE13 | Female | 39 | 6 |
| SLE14 | Female | 22 | 4 |
| SLE15 | Female | 30 | 6 |
| SLE16 | Female | 60 | 3 |
| SLE17 | Female | 29 | 4 |
| SLE18 | Female | 21 | 8 |
| SLE19 | Female | 42 | 12 |
| SLE20 | Female | 42 | 4 |
| SLE21 | Female | 15 | 8 |
| SLE22 | Female | 70 | 3 |
| SLE23 | Female | 34 | 4 |
| SLE24 | Female | 51 | 6 |
| SLE25 | Female | 63 | 4 |
| SLE26 | Female | 54 | 6 |
| SLE27 | Female | 50 | 6 |
| SLE28 | Female | 59 | 8 |
| SLE29 | Female | 25 | 14 |
| SLE30 | Female | 57 | 4 |
| SLE31 | Female | 55 | 4 |
| SLE32 | Male | 51 | 2 |
| SLE33 | Female | 56 | 2 |
| SLE34 | Female | 54 | 6 |
| SLE35 | Female | 37 | 8 |
| SLE36 | Female | 33 | 12 |
| SLE37 | Female | 40 | 4 |
| SLE38 | Female | 25 | 8 |
| SLE39 | Female | 55 | 4 |
| SLE40 | Female | 64 | 2 |
| SLE41 | Female | 42 | 8 |
| SLE42 | Female | 53 | 2 |
| SLE43 | Female | 35 | 12 |
| SLE44 | Female | 37 | 6 |
| PS1 | Male | 34 | NA |
| PS2 | Male | 55 | NA |
| PS3 | Male | 56 | NA |
| PS4 | Male | 67 | NA |
| PS5 | Male | 30 | NA |
| PS6 | Female | 72 | NA |
| PS7 | Male | 49 | NA |
| PS8 | Female | 20 | NA |
| PS9 | Male | 32 | NA |
| PS10 | Male | 50 | NA |
| PS11 | Female | 37 | NA |
| PS12 | Male | 45 | NA |
| PS13 | Male | 31 | NA |
| PS14 | Male | 21 | NA |
| PS15 | Female | 34 | NA |
| PS16 | Male | 55 | NA |
| PS17 | Male | 42 | NA |
| PS18 | Male | 34 | NA |
| PS19 | Male | 53 | NA |
| PS20 | Male | 60 | NA |
| PS21 | Male | 43 | NA |
| PS22 | Female | 36 | NA |
| PS23 | Male | 50 | NA |
| PS24 | Female | 18 | NA |
| RA1 | Female | 50 | NA |
| RA2 | Female | 48 | NA |
| RA3 | Female | 40 | NA |
| RA4 | Female | 53 | NA |
| RA5 | Female | 63 | NA |
| RA6 | Female | 55 | NA |
| RA7 | Female | 71 | NA |
| RA8 | Male | 55 | NA |
| RA9 | Female | 53 | NA |
| RA10 | Female | 28 | NA |
| RA11 | Female | 59 | NA |
| RA12 | Female | 48 | NA |
| RA13 | Female | 47 | NA |
| RA14 | Female | 59 | NA |
| RA15 | Male | 62 | NA |
| RA16 | Male | 44 | NA |
| RA17 | Female | 29 | NA |
| RA18 | Female | 54 | NA |
| RA19 | Female | 66 | NA |
| RA20 | Female | 59 | NA |
| RA21 | Female | 49 | NA |
| RA22 | Female | 58 | NA |
| RA23 | Female | 67 | NA |
| RA24 | Male | 53 | NA |
| pSS1 | Female | 38 | NA |
| pSS2 | Female | 42 | NA |
| pSS3 | Male | 52 | NA |
| pSS4 | Female | 50 | NA |
| pSS5 | Female | 48 | NA |
| pSS6 | Female | 39 | NA |
| pSS7 | Female | 55 | NA |
| pSS8 | Female | 27 | NA |
| pSS9 | Female | 45 | NA |
| pSS10 | Female | 34 | NA |
| pSS11 | Female | 32 | NA |
| pSS12 | Male | 46 | NA |
| pSS13 | Female | 39 | NA |
| pSS14 | Female | 28 | NA |
| pSS15 | Female | 72 | NA |
| pSS16 | Female | 56 | NA |
| pSS17 | Female | 47 | NA |
| pSS18 | Female | 56 | NA |
| pSS19 | Female | 36 | NA |
| pSS20 | Female | 43 | NA |
| pSS21 | Female | 46 | NA |
| pSS22 | Female | 20 | NA |
| pSS23 | Female | 49 | NA |
| pSS24 | Female | 64 | NA |
| HC1 | Female | 56 | NA |
| HC2 | Female | 54 | NA |
| HC3 | Female | 48 | NA |
| HC4 | Female | 42 | NA |
| HC5 | Female | 46 | NA |
| HC6 | Female | 52 | NA |
| HC7 | Female | 49 | NA |
| HC8 | Female | 50 | NA |
| HC9 | Female | 25 | NA |
| HC10 | Male | 26 | NA |
| HC11 | Female | 22 | NA |
| HC12 | Female | 23 | NA |
| HC13 | Female | 29 | NA |
| HC14 | Female | 29 | NA |
| HC15 | Female | 28 | NA |
| HC16 | Male | 25 | NA |
| HC17 | Male | 27 | NA |
| HC18 | Female | 38 | NA |
| HC19 | Female | 25 | NA |
| HC20 | Female | 27 | NA |
| HC21 | Female | 29 | NA |
| HC22 | Female | 26 | NA |
| HC23 | Female | 30 | NA |
| HC24 | Female | 36 | NA |

SLE1-SLE20 were recruited for METTL3 and SLEDAI score correlation analysis, SLE21-SLE44 were enrolled for comparing METTL3 expression. SLE: Systemic lupus erythematosus; SLEDAI: SLE disease activity index; PS: psoriasis; RA: Rheumatoid arthritis; pSS: primary Sjogren's syndrome; HC: Healthy control; NA: Not applicable.
