# Supplementary material for: m6A methyltransferase METTL3 programs CD4+ T-cell activation and effector T-cell differentiation in systemic lupus erythematosus
Source: Mol Med. 2023 Apr 3;29:46. doi: 10.1186/s10020-023-00643-4 (PMC10068720; doi:10.1186/s10020-023-00643-4)
Supplement: Supplementary file 2 — Additional file 2: Table S2. Information on materials and reagents for Flow analysis. [file 10020_2023_643_MOESM2_ESM.docx]

**Table S2. Reagents for Flow cytometry analysis**

| Reagent | Source | Identifier |
| --- | --- | --- |
| Zombie NIR^TM^ Fixable Viability Kit | Biolegend | 423106 |
| FITC Mouse Anti-Human CD4 | BD Pharmingen | 555346 |
| PE-Cyanine7 Mouse Anti-Human CXCR5 | eBioscience | 25-9185-42 |
| APC Mouse Anti-Human PD-1 | Biolegend | 329908 |
| PE Mouse Anti-Human CD25 | eBioscience | 12-0259-42 |
| APC Mouse Anti-Human FOXP3 | eBioscience | 17-4776-42 |
| FITC Mouse Anti-Human CD69 | BD Pharmingen | 555530 |
| APC Mouse Anti-Human CD4 | Biolegend | 300552 |
| FITC Rat Anti-Mouse CD4 | Biolegend | 100406 |
| PE-Cy^TM^7 Rat Anti-Mouse CXCR5 | BD Pharmingen | 560617 |
| APC Hamster Anti-Mouse PD-1 | BD Pharmingen | 551892 |
| APC Rat Anti-Mouse FOXP3 | eBioscience | 17-4776-42 |
| FITC Rat Anti-Mouse T-and B-Cell Activation Antigen (GL-7) | BD Pharmingen | 553666 |
| APC Rat Anti-Mouse B220 | BD Pharmingen | 553092 |
| PE/Cyanine7 Anti-Mouse CD138 | Biolegend | 142514 |
| APC Rat Anti-Mouse IL-4 | eBioscience | 17-7041-82 |
| APC-Cy^TM^7 Rat Anti-Mouse IL-17A | BD Pharmingen | 560821 |
| PerCP-Cy^TM^5.5 Rat Anti-Mouse IFN-γ | BD Pharmingen | 560660 |
| Cytofix/Cytoperm^TM^ Fixation/Permeabilization Solution Kit | BD Pharmingen | 554714 |
| Foxp3/Transcription Factor Staining Buffer Set | eBioscience | 00-5523-00 |
| Leukocyte Activation Cocktail, with BD GolgiPlμg^TM^ | BD Pharmingen | 550583 |
| PE-Cy^TM^7 Rat Anti-Mouse CD44 | BD Pharmingen | 560569 |
| PerCP/Cyanine5.5 Anti-Mouse CD8a | Biolegend | 100734 |
| APC-Cy^TM^7 Rat Anti-Mouse CD62L | BD Pharmingen | 560514 |
| APC Hamster Anti-Mouse CD3e | BD Pharmingen | 561826 |
| PE-Hamster Anti-Mouse Fas | BD Pharmingen | 554258 |
| APC Rat Anti-Mouse CD127 | BD Pharmingen | 564175 |
