# Supplementary material for: m6A methyltransferase METTL3 programs CD4+ T-cell activation and effector T-cell differentiation in systemic lupus erythematosus
Source: Mol Med. 2023 Apr 3;29:46. doi: 10.1186/s10020-023-00643-4 (PMC10068720; doi:10.1186/s10020-023-00643-4)
Supplement: Supplementary file 3 — Additional file 3: Table S3. Sequence information on siRNA and primers [file 10020_2023_643_MOESM3_ESM.docx]

**Table S3. Sequence information on siRNA and primers**

| Target | Sequence |
| --- | --- |
| ALKBH5 | Forward: 5’-3’ TCGGCGAAGGCTACACTTAC |
|  | Reverse: 5’-3’ TGGTAGTCGTTGATGACGGC |
| FTO | Forward: 5’-3’ TGGGTTCATCCTACAACGG |
|  | Reverse: 5’-3’ CCTCTTCAGGGCCTTCAC |
| GAPDH | Forward: 5’-3’ GGAGCGAGATCCCTCCAAAAT |
|  | Reverse: 5’-3’ GGCTGTTGTCATACTTCTCATGG |
| ACTB | Forward: 5’-3’ CATGTACGTTGCTATCCAGGC |
|  | Reverse: 5’-3’ CTCCTTAATGTCACGCACGAT |
| NFATC2 | Forward: 5’-3’ ACGAGCTTGACTTCTCCATCC |
|  | Reverse: 5’-3’ AGAAACTTCTGCGGCCCTAC |
| mTOR | Forward: 5’-3’ CGCGAACCTCAGGGCAA |
|  | Reverse: 5’-3’ ATTCCGGCTCTTTAGGCCAC |
| CBFB | Forward: 5’-3’ GAAAGAGAAGCAGGCAAGGTATATT |
|  | Reverse: 5’-3’ CTTGTTGTCTTCTTGCCTCCA |
| TNFRSF18 | Forward: 5’-3’ GAAGGCCACTGCAAACCTTG |
|  | Reverse: 5’-3’ ACATGCACTGACTCCTCAGC |
| CTLA4 | Forward: 5’-3’ CATGTACCCACCGCCATACT |
|  | Reverse: 5’-3’ CCCGAACTAACTGCTGCAAG |
| HK2 | Forward: 5’-3’ TGTGAATCGGAGAGGTCCCA |
|  | Reverse: 5’-3’ AACTTCGGCCACAGGATCAC |
| SLC7A11 | Forward: 5’-3’ GGACAAGAAACCCAGGTGGT |
|  | Reverse: 5’-3’ GCAGATTGCCAAGATCTCAAGT |
| TREML2 | Forward: 5’-3’ TACAAAAACCGCGTGGAGGG |
|  | Reverse: 5’-3’ GTTGCGCATGCACCAGTATC |
| Foxp3 | Forward: 5’-3’ GGCACAATGTCTCCTCCAGA |
|  | Reverse: 5’-3’ CTTGTCGGATGATGCCACAG |
| METTL3 | Forward: 5’-3’ TGAGGTAAAGCGAGGTCTCC |
|  | Reverse: 5’-3’ TGGTTCCGATGCTGAAGAGT |
| METTL14 | Forward: 5’-3’ TTGCAGCACCTCGATCATTT |
|  | Reverse: 5’-3’ TGAATGAAGTCCCCGTCTGT |
| Scramble siRNA | Sense: 5’-3’ AAGAGGCTTGCACAGTGCA |
|  | Antisense: 5’-3’ TGCACTGTGCAAGCCTCTT |
| METTL3 siRNA pool | Sense: 5’-3’ ACTTCTTCTCTAATTCAGGGT |
|  | Antisense: 5’-3’ ACCCTGAATTAGAGAAGAAGT |
|  | Sense: 5’-3’ CCTGCAAGTATGTTCACTA |
|  | Antisense: 5’-3’ TAGTGAACATACTTGCAGG |
|  | Sense: 5’-3’ GCTACCTGGACGTCAGTAT |
|  | Antisense: 5’-3’ ATACTGACGTCCAGGTAGC |
| Foxp3 1# | Forward: 5’-3’ CTTCCTTGAACCCCATGCCA |
|  | Reverse: 5’-3’ GCATGAAATGTGGCCTGTCC |
| Foxp3 2# | Forward: 5’-3’ CACTGCTGGCAAATGGTGTC |
|  | Reverse: 5’-3’ GCTGCTCCAGAGACTGTACC |
| Foxp3 3# | Forward: 5’-3’ CCCAGAGTTCCTCCACAACA |
|  | Reverse: 5’-3’ TCATTGAGTGTCCGCTGCTT |
