# Supplementary material for: m6A methyltransferase METTL3 programs CD4+ T-cell activation and effector T-cell differentiation in systemic lupus erythematosus
Source: Mol Med. 2023 Apr 3;29:46. doi: 10.1186/s10020-023-00643-4 (PMC10068720; doi:10.1186/s10020-023-00643-4)
Supplement: Supplementary file 4 — Additional file 4: Table S4. Antibody information for Western blot and ELISA [file 10020_2023_643_MOESM4_ESM.docx]

**Table S4. Antibody information for Western blot and ELISA**

| Target | Source | Dilution |
| --- | --- | --- |
| FTO | Abcam, ab126605 | 1:1000 |
| ALKBH5 | Abcam, ab195377 | 1:1000 |
| METTL3 | Abcam, ab195352 | 1:1000 |
| METTL14 | Proteintech, 26158-1-AP | 1:1000 |
| WTAP | Abcam, ab195380 | 1:1000 |
| Foxp3 | Proteintech, 22228-1-AP | 1:1000 |
| BRD4 | Proteintech, 67374-2-Ig | 1:1000 |
| MGMT | Proteintech, 17195-1-AP | 1:1000 |
| β-actin | Proteintech, 66009-1-Ig | 1:1000 |
| GAPDH | Proteintech, 60004-1-Ig | 1:1000 |
| IgM | Bethyl Laboratories, A90-101P | 1:3000 |
| IgG1 | Bethyl Laboratories, A90-105P | 1:3000 |
| IgG2a | Bethyl Laboratories, A90-107P | 1:3000 |
| IgG2b | Bethyl Laboratories, A90-109P | 1:5000 |
| IgG (H+L) | Bethyl Laboratories, A90-116P | 1:3000 |
| IgG3 | Bethyl Laboratories, A90-111P | 1:3000 |
