# Supplementary figures and images for: m6A methyltransferase METTL3 programs CD4+ T-cell activation and effector T-cell differentiation in systemic lupus erythematosus
Source: Mol Med. 2023 Apr 3;29:46. doi: 10.1186/s10020-023-00643-4 (PMC10068720; doi:10.1186/s10020-023-00643-4)

Original blots


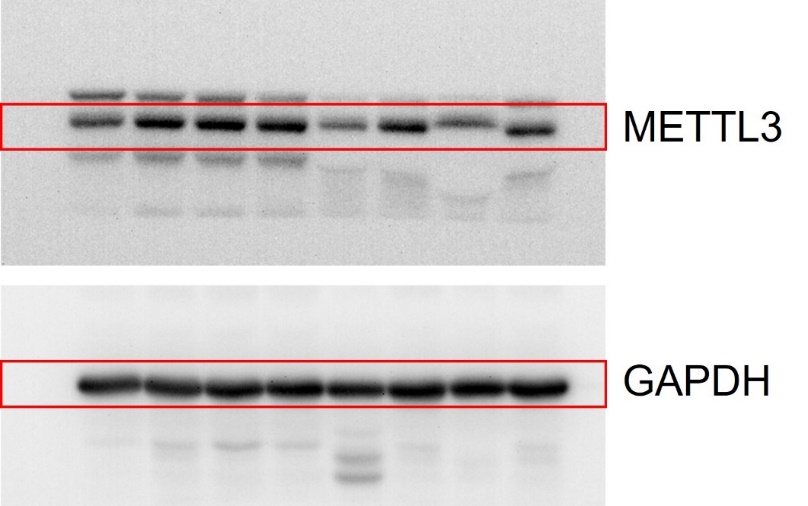


Fig. 1b


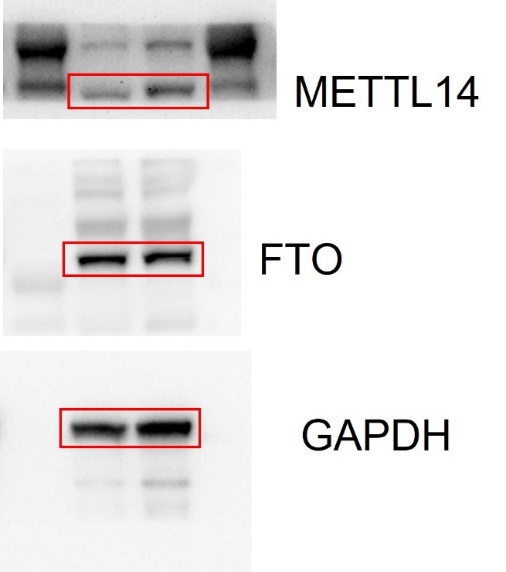


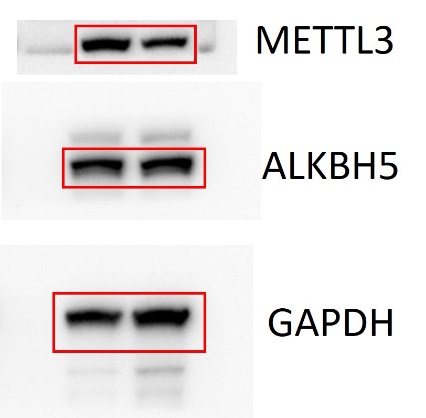


Fig. 2a


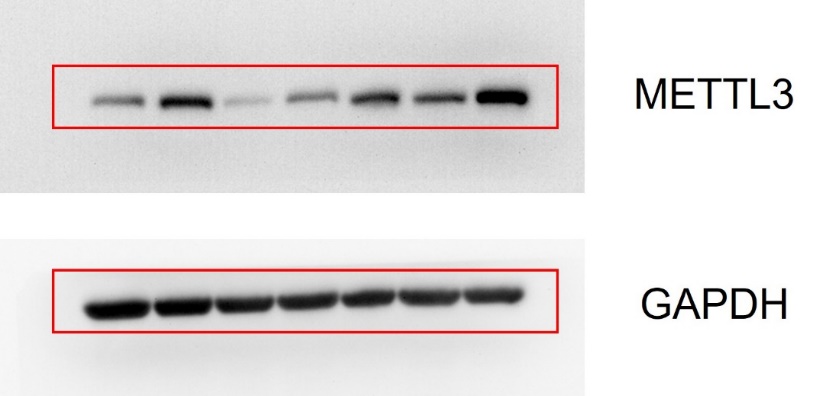


Fig.2c


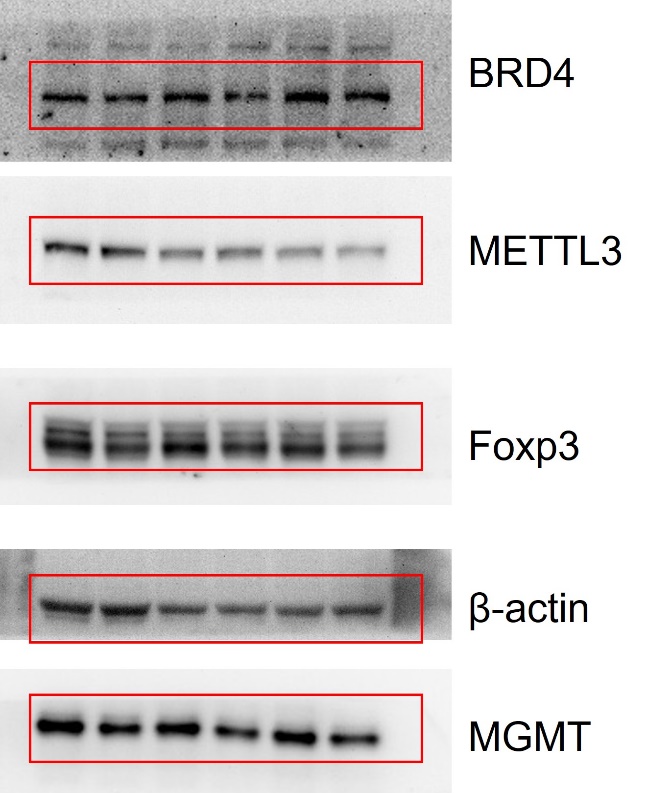


Fig. 6a


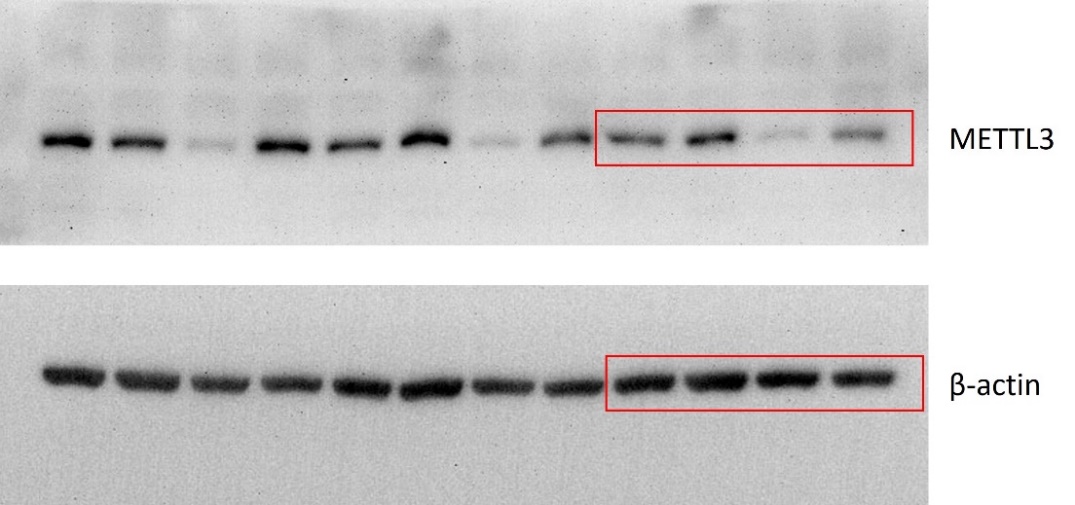


Fig. S1c


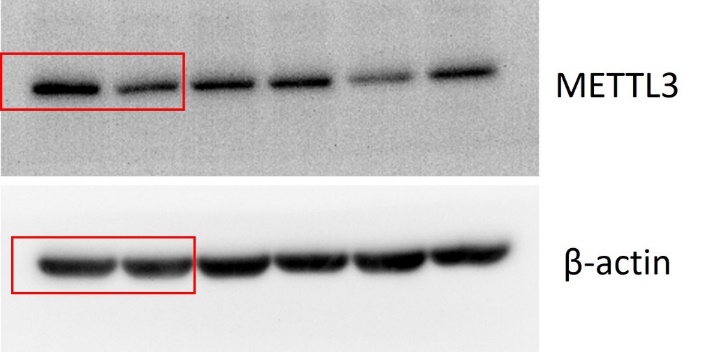


Fig. S4c

Supplement: Supplementary file 5 — Additional file 5. Original blots of Western blot analysis. [file 10020_2023_643_MOESM5_ESM.docx]
