# Supplementary material for: m6A methyltransferase METTL3 programs CD4+ T-cell activation and effector T-cell differentiation in systemic lupus erythematosus
Source: Mol Med. 2023 Apr 3;29:46. doi: 10.1186/s10020-023-00643-4 (PMC10068720; doi:10.1186/s10020-023-00643-4)
Supplement: Supplementary file 6 — Additional file 6: Figure S1. METTL3 expression varies after T-cell activation and Treg-cell differentiation in vitro. a Representative dot plots showing the proportions of CD4+ T cells before and after activation by anti-CD3 and anti-CD28 antibodies for 3 days. b The mRNA expression of METTL3 in CD4+ T cells activated by anti-CD3 and anti-CD28 antibodies at 0, 12, 48, and 72 h was detected by RT-qPCR, n = 3. c Left: Western blot of METTL3 expression in Th0 and differentiated Tregs at 3 and 5 days, and β-actin was used as a loading control; right: quantification of METTL3 protein expression in Th0 and differentiated Tregs, n = 3. (***p < 0.001, ns, no significance, one-way ANOVA with Dunnett’s multiple comparisons test for b, two-way ANOVA with Sidak’s multiple comparisons test for c). Figure S2. Anti-SRBC antibodies and splenic Tfh cells are significantly elevated in SRBC-immunized mice. a ELISA of anti-SRBC specific antibodies IgM, IgG1, IgG2a, IgG2b, IgG (H+L), and IgG3 in the blood serum of control mice and SRBC-immunized mice. Serum dilution: IgM: 1:200; IgG1, IgG2a, and IgG (H+L): 1:400; IgG2b: 1:800; IgG3: 1:40; n = 6. b Left: representative dot plots showing the proportion of splenic CD4+ T cells in mice after SRBC challenge; right: quantification of splenic CD4+PD1+CXCR5+ Tfh cells, n = 6. (*p < 0.05, **p < 0.01, ***p < 0.001 ****p < 0.0001, unpaired two-tailed Student’s t test). Figure S3. Proportions of Tfh and Th2 cells remain comparable between control and STM2457-administered cGVHD mice. a Comparison of the body weights of cGVHD mice body treated with DMSO or STM2457. b Left: spleen and dLN images of cGVHD mice; right: quantification of spleen weight. c Top: representative dot plots showing the proportion of CD4+ T cells in the spleen and dLNs of cGVHD mice treated with DMSO or STM2457; bottom: quantification of CD4+PD1+CXCR5+ Tfh cells. d Left: representative dot plots showing the proportion of splenic CD4+ T cells in the spleens of cGVHD mice treated [file 10020_2023_643_MOESM6_ESM.docx]

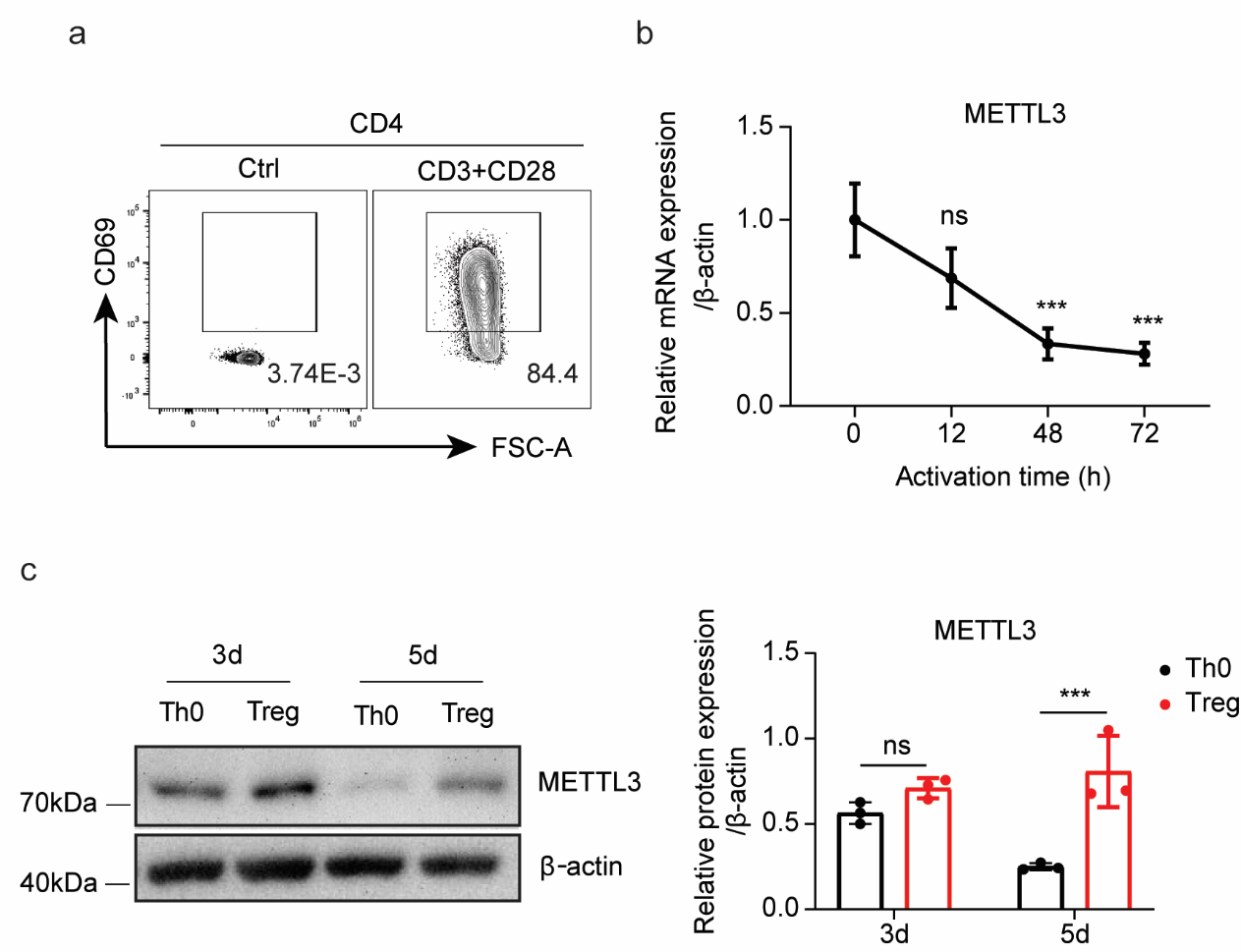


**Figure S1 METTL3 expression varies after T-cell activation and Treg-cell differentiation *in vitro*. a** Representative dot plots showing the proportions of CD4^+^ T cells before and after activation by anti-CD3 and anti-CD28 antibodies for 3 days. **b** The mRNA expression of METTL3 in CD4^+^ T cells activated by anti-CD3 and anti-CD28 antibodies at 0, 12, 48, and 72 hours was detected by RT-qPCR, n=3. **c** Left: Western blot of METTL3 expression in Th0 and differentiated Tregs at 3 and 5 days, and β-actin was used as a loading control; right: quantification of METTL3 protein expression in Th0 and differentiated Tregs, n=3. (****p*<0.001, ns, no significance, one-way ANOVA with Dunnett’s multiple comparisons test for **b**, two-way ANOVA with Sidak’s multiple comparisons test for **c**.)


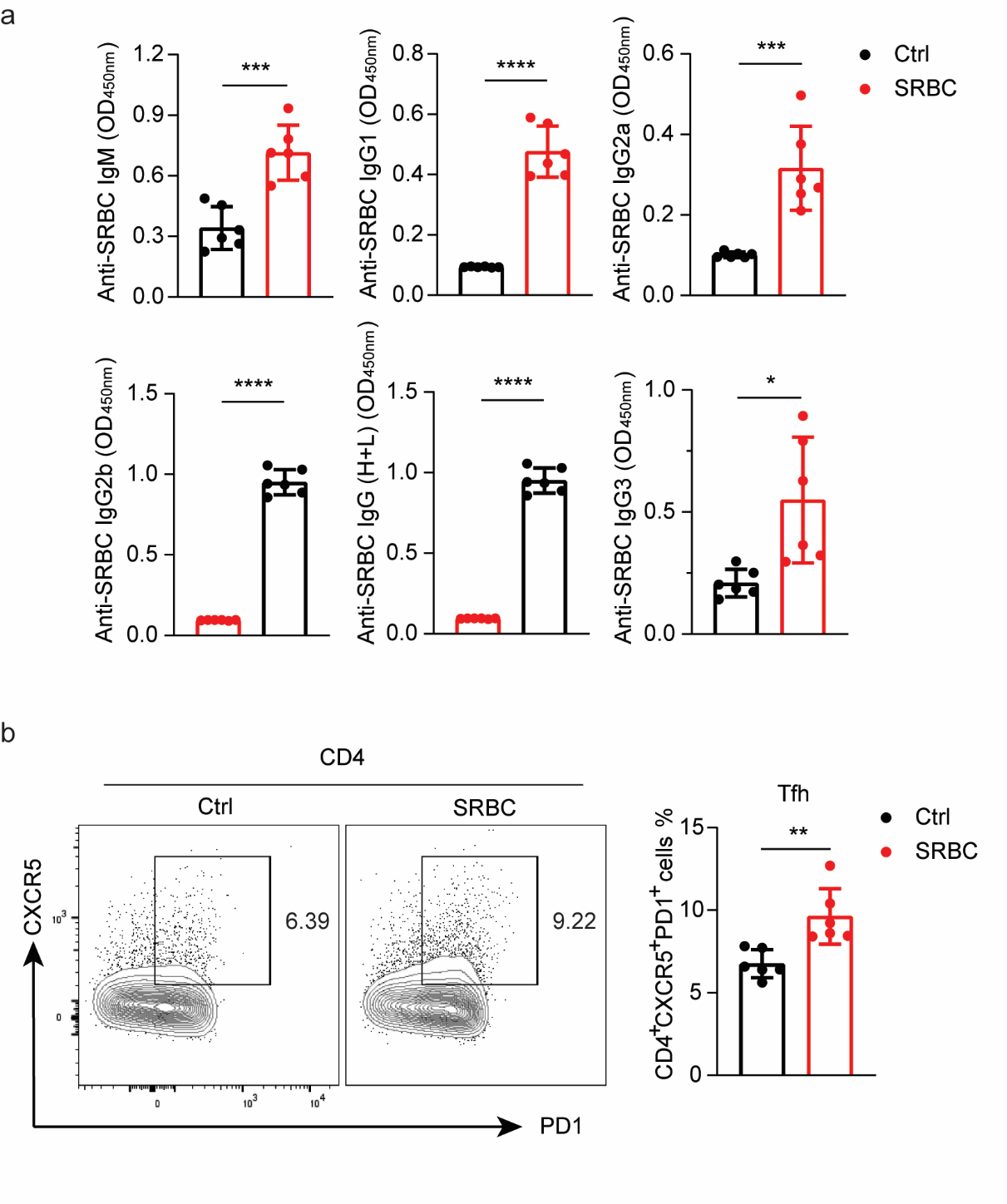


**Figure S2 Anti-SRBC antibodies and splenic Tfh cells are significantly elevated in SRBC-immunized mice.** **a** ELISA of anti-SRBC specific antibodies IgM, IgG1, IgG2a, IgG2b, IgG (H+L), and IgG3 in the blood serum of control mice and SRBC-immunized mice. Serum dilution: IgM: 1:200; IgG1, IgG2a, and IgG (H+L): 1:400; IgG2b: 1:800; IgG3: 1:40; n=6. **b** Left: representative dot plots showing the proportion of splenic CD4^+^ T cells in mice after SRBC challenge; right: quantification of splenic CD4^+^PD1^+^CXCR5^+^ Tfh cells, n=6. (**p*<0.05, ***p*<0.01, ****p*<0.001 *****p*<0.0001, unpaired two-tailed Student’s *t* test.) **
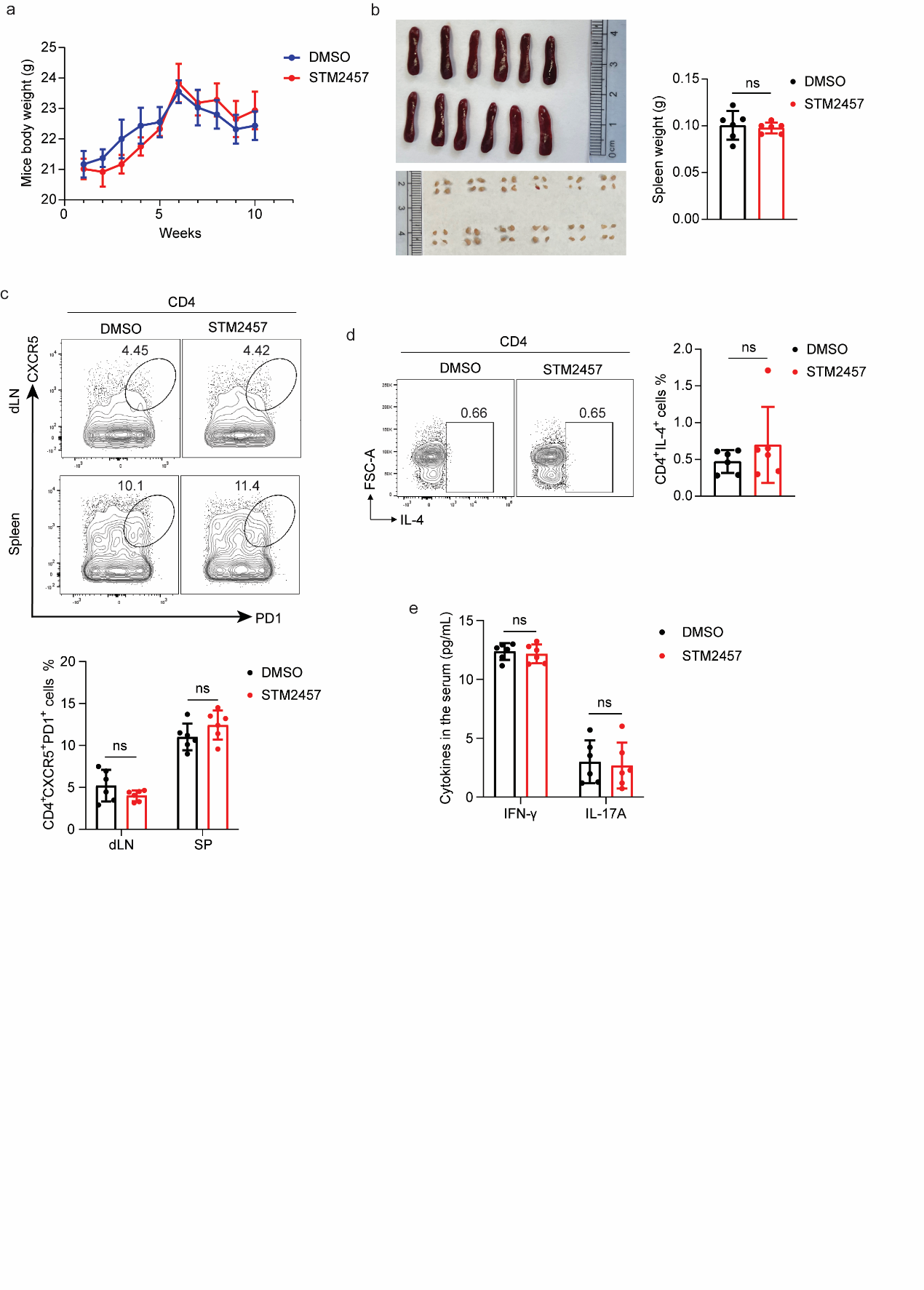
**

**Figure S3 Proportions of Tfh and Th2 cells remain comparable between control and STM2457-administered cGVHD mice. a** Comparison of the body weights of cGVHD mice body treated with DMSO or STM2457. **b** Left: spleen and dLN images of cGVHD mice; right: quantification of spleen weight. **c** Top: representative dot plots showing the proportion of CD4^+^ T cells in the spleen and dLNs of cGVHD mice treated with DMSO or STM2457; bottom: quantification of CD4^+^PD1^+^CXCR5^+^ Tfh cells. **d** Left: representative dot plots showing the proportion of splenic CD4^+^ T cells in the spleens of cGVHD mice treated with DMSO or STM2457; right: quantification of CD4^+^IL-4^+^ Th2 cells. e ELISA of IFN-γ and IL-17A levels in the blood serum of cGVHD mice treated with DMSO or STM2457. (ns, no significance, unpaired two-tailed Student’s *t* test.) **
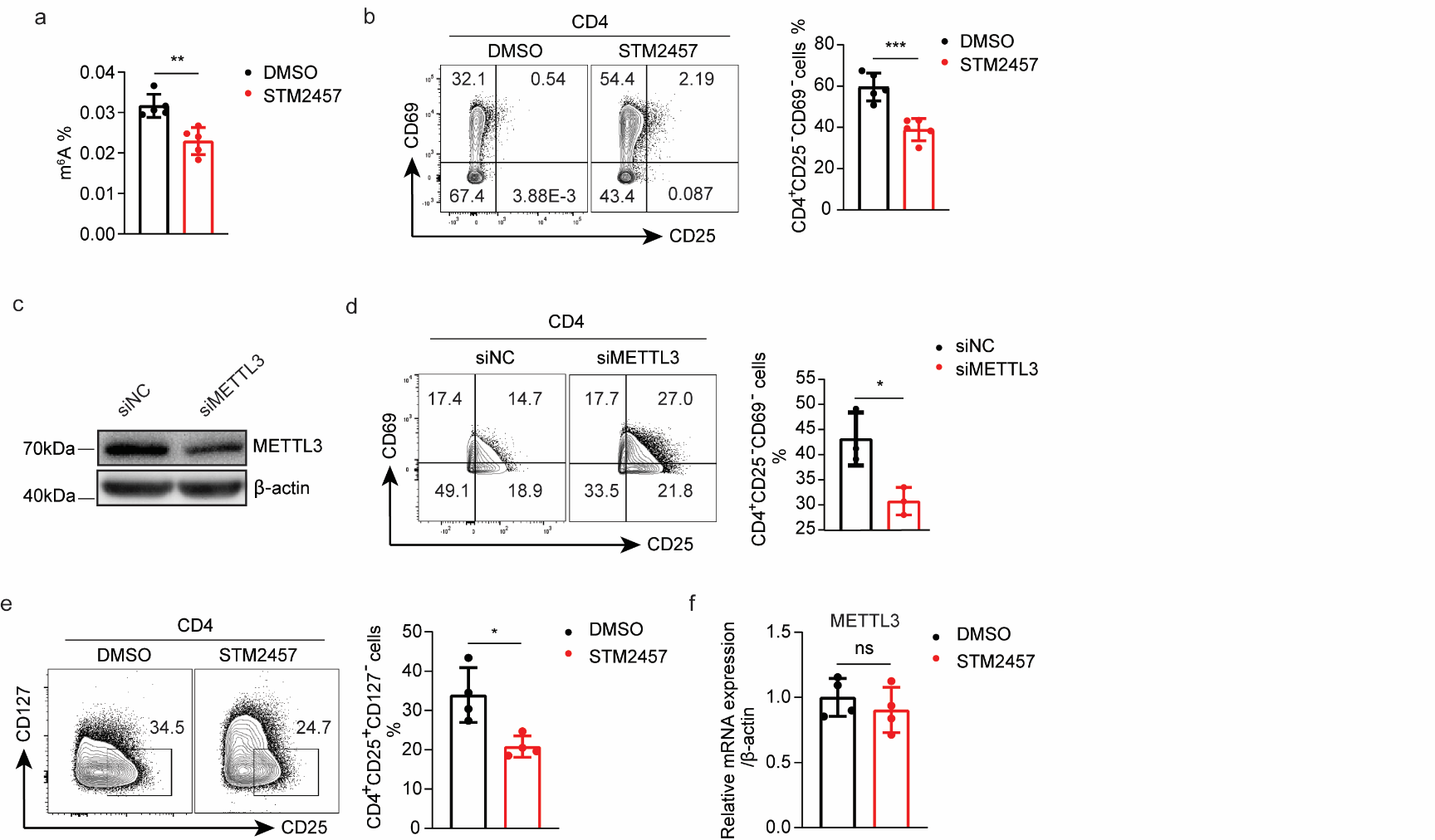
**

**Figure S4 METTL3 catalytic inhibition or gene knockdown suppresses CD4^+^ T-cell activation *in vitro*. a** Comparison of total m^6^A modification between STM2457- and DMSO-treated CD4^+^ T cells by colorimetric quantification, n=5. **b** Left: representative dot plots showing the composition of CD4^+^ T cells in STM2457 (5 μM)-treated and DMSO control cells after activation by anti-CD3 and anti-CD28 antibodies for 3 days; right: quantification of nonactivated CD4^+^CD25^-^CD69^-^ cells, n=5. **c** Immunoblot of METTL3 in CD4^+^ T cells treated with either METTL3 siRNA or scramble siRNA, and β-actin was used as a loading control. **d** Left: representative dot plots showing the proportion of CD4^+^ T cells treated with either scramble siRNA or METTL3 siRNA for 3 days after being activated by anti-CD3 and anti-CD28 antibodies; right: quantification of unactivated CD4^+^CD25^-^CD69^-^ cells, n=3. e Left: representative dot plots showing the proportion of CD4^+^ T cells treated with either DMSO or STM2457 for 5 days when cultured under Treg differentiation conditions; right: quantification of CD4^+^CD25^+^CD127^-^ cells, n=4. **f** The mRNA expression of METTL3 in SLE CD4^+^ T cells treated with either DMSO or STM2457 when cultured under Treg differentiation conditions was detected by RT-qPCR, n=4. (**p*<0.05, ***p*<0.01, ****p*<0.001, ns, no significance, unpaired two-tailed Student’s *t* test.)
